# 27th Annual GP2A Medicinal Chemistry Conference

**DOI:** 10.3390/ph12040179

**Published:** 2019-12-06

**Authors:** Shailesh N. Mistry, Pascal Marchand, Barrie Kellam

**Affiliations:** 1 School of Pharmacy, Centre for Biomolecular Sciences, University of Nottingham, Nottingham NG7 2RD, UK; barrie.kellam@nottingham.ac.uk; 2 Université de Nantes, Cibles et Médicaments des Infections et du Cancer, IICiMed, EA 1155, F-44000 Nantes, France

**Keywords:** medicinal chemistry, drug design, chemical biology, ligand kinetics

## Abstract

The 27th annual GP2A (Groupement des Pharmacochimistes de l′Arc Atlantique/Group of Medicinal Chemists in the Atlantic Arc) conference took place from 21 to 23 August 2019, at the East Midlands Conference Centre (University Park, Nottingham, United Kingdom) and was hosted by the Division of Biomolecular Science and Medicinal Chemistry (BSMC), within the School of Pharmacy at the University of Nottingham. The event brought together an international delegation of researchers with interests in medicinal chemistry and interfacing disciplines. In addition, a pre-conference workshop provided an opportunity for younger researchers to develop their theoretical knowledge in quantitative pharmacology. Abstracts of presentations by the 14 invited speakers and 6 young researchers, in addition to 41 posters, are included in this report.

## 1. Aim and Scope of the Meeting

The GP2A (Groupement des Pharmacochimistes de l′Arc Atlantique/Group of Medicinal Chemists in the Atlantic Arc) network is made up of researchers in the field of medicinal chemistry working in universities and research institutes across Europe. It was founded in 1992 with the aim of bringing together researchers to exchange ideas and expertise and facilitate a friendly collaborative networking environment. Historically, it has included members from France, Spain, Portugal, Ireland and the United Kingdom (the “Atlantic Arc”). More recently, it has expanded both geographically and in terms of research fields, now including researchers from areas that range from physical and pharmaceutical chemistry to molecular pharmacology.

The annual GP2A conference host city alternates between France and another country and brings together established scientists and those earlier in their career. In addition, the network facilitates short visits between laboratories. A consistent theme of the annual conference is to provide young researchers an opportunity to present their work to an international audience through either poster presentation or by sharing the stage with invited speakers.

The 2019 conference was held at the East Midlands Conference Centre and hosted by the Division of Biomolecular Science and Medicinal Chemistry (BSMC), within the School of Pharmacy at the University of Nottingham (UoN). Ahead of the workshop, young researchers were invited to attend a pre-conference workshop, led by Prof. Steven J. Charlton (UoN/Excellerate Bioscience) and Dr Elizabeth Rosethorne (UoN). The meeting opened and closed with two internationally recognised plenary speakers, Prof. Peter Gmeiner (Friedrich-Alexander University, Erlangen-Nürnberg, Germany) and Prof. Rob Leurs (Vrije-Universiteit, Amsterdam, The Netherlands). In addition, there were a further 12 invited speaker talks, 6 young researcher talks and 41 posters, selected from over 50 submitted abstracts.

Reflective of the multidisciplinary nature of medicinal chemistry research, the meeting included a broad range of topics of interest. These included infectious and neurodegenerative diseases, chemoprevention of cancer, approaches to target identification, hit identification and optimisation for drug candidates, natural products, inflammatory diseases/pain and the application of structural cheminformatics.

## 2. Pre-Conference Workshop on Quantitative Pharmacology

Comparing the pharmacological characteristics of compounds across different systems (e.g., different assays and/or cell types) can be a complex task. More difficult still is the extrapolation of these in vitro data to in vivo systems through PK/PD modelling. In order to make these tasks as accurate as possible, it is critical to define quantitative, system independent measures of compound action.

This workshop will introduce the concept of quantitative molecular pharmacology and detail the experimental and analytical methods used to determine key system-independent pharmacological descriptors of both drug binding and functional efficacy. It will culminate in a team-based exercise where you will put the knowledge and skills gained from the morning′s lectures into practice. You will play the role of the pharmacologist in a (fictitious) pharmaceutical company′s due diligence team, tasked with identifying the best compound to in-license for a respiratory indication. These deals can be very expensive—will you be confident enough in your analysis to make a recommendation to senior management…?

## 3. Opening Plenary Lecture

### Selective Ligands Based on GPCR Structures

GmeinerPeterDepartment of Chemistry and Pharmacy, Medicinal Chemistry, Emil Fischer Center, Friedrich-Alexander University, Nikolaus-Fiebiger- Str. 10, 91058 Erlangen, Germany; peter.gmeiner@fau.de

GPCR crystal structures may leverage an effective development of novel drug candidates because they can be used for structure-based in silico docking screens, giving access to new chemotypes and, as a consequence, to new biological profiles (Shoichet, B.K., Kobilka, B.K. *Trends Pharmacol. Sci.* **2012**, *33*, 268–272). Furthermore, they can guide lead modification and optimization programs by providing insights into crucial ligand–receptor interactions and the bioactive ligand conformation. Both strategies can be performed on the basis of either the crystal structure of a given GPCR or from a homology model of a structurally highly similar congener. Structures of different activity states of GPCRs allow us to identify molecular interactions discriminating between inverse agonists, antagonists and agonists. These fundamental results also contribute to the rational discovery of drugs selectively binding to particular conformational states.

We aim to leverage GPCR structures to discover new chemotypes and bioisosteres with beneficial physical and biological properties and use these to probe and control signalling pathways. Thus, we develop highly specific agonists and antagonists for Class A GPCRs for structural and functional investigations and for structure–function relationship studies. The final goal is the development of novel drug candidates. 

The seminar will show very recent developments in the design, synthesis and biological investigation of orthosteric and allosteric GPCR ligands with unprecedented activity profiles.


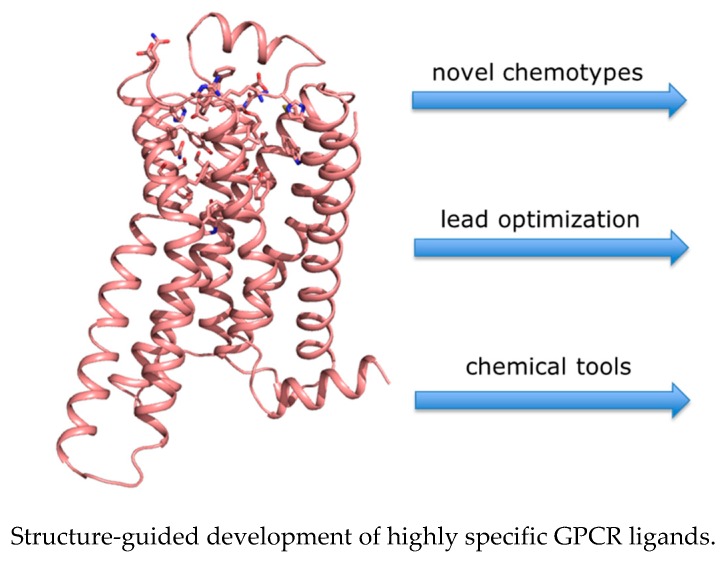


## 4. Closing Plenary Lecture

### It is All About Time…..

LeursRobDepartment of Medicinal Chemistry, Vrije Universiteit Amsterdam, 1081 HZ Amsterdam, The Netherlands; r.leurs@vu.nl

In light of the recent interest in drug binding kinetics, we have investigated the kinetics of the binding interactions between the classical antihistamines and the H1R within the framework of the Innovative Medicines Initiative-sponsored program “Kinetics for Drug Discovery (K4DD)”. We developed new methodologies to measure and study GPCR-ligand kinetics and investigated the relationship between the drug structure and the drug-receptor binding kinetics (i.e., structure kinetics relationship, SKR). A better understanding of this relationship should provide a handle for the rational drug optimization of the kinetic binding profile of antihistamines.

Large differences in H1R binding kinetics were observed between different antihistamines that were not always reflected by the strength of the binding interaction (affinity). It was additionally shown that differences in the binding kinetics of antihistamines at the H1R affect their mode of antagonism of the GPCR, independent of their binding affinity. For ligands binding the H1R, several sub-structures were linked to an increase in the receptor residence time. Moreover, several of the newer generation antihistamines (e.g., levocetirizine, olopatadine, rupatadine) contained one or multiple of these chemical structures, explaining their long residence time at the H1R and most likely also their therapeutic success.

This, together with evidence presented in the scientific literature, suggests that kinetic analyses of drug-receptor binding have added value for the development of new drugs. Focus on a better understanding of the molecular interactions that are formed between the drug and receptor over time and the relationship with ligand binding rate constants are therefore a step forward in the development of effective GPCR ligands.

## 5. Keynote Lectures

### 5.1. The Design and Development of Keap1-Nrf2 Interaction Inhibitors and Their Biological Activity (KL1)

GeorgakopoulosNikolaousGatliffJemmaWellsGeoffUCL School of Pharmacy, University College London, London WC1N 1AX, UK; g.wells@ucl.ac.uk

The therapeutic potential of Nrf2-inducing molecules spans several disease states including chronic neurodegenerative diseases, inflammatory conditions and possible roles in cancer chemoprevention. Conversely, targeted use of Nrf2 inhibitors may have applications in cancer treatment regimens (Cuadrado, A., et al. *Nat. Rev. Drug Disc.* **2019**, *18*, 295–317).

Our research has been directed towards identifying both reversible and irreversible inhibitors of the Keap1-Nrf2 protein–protein interaction. Reactive inducers have been developed from a previous class of multi-targeted redox homeostasis modulators. On the other hand, reversible inhibitors of Keap1 are the product of structure-based design utilising peptide and small molecule leads. We have identified high affinity sulphonamide ligands for Keap1 (IC_50_ < 1 nM) and compounds with non-standard binding modes utilising X-ray crystallography studies.

In our analysis of the biological activity of these compounds, we characterised effects on mitochondrial function and quality control processes, and autophagy that differ between reversible and irreversible Nrf2 inducers. Differences in mitochondrial turnover, ROS and biogenesis appear to distinguish the two types of Nrf2 inducer across several distinct chemotypes.

The activity of these small molecules can contribute to understanding the biological activities of different classes of Nrf2 inducers, their therapeutic utility and potential off-target effects and toxicity.

### 5.2. Novel Antikinetoplastid Nitroaromatics Bioactivated by Type 1 NTRs (KL2)

VerhaegheP.Faculté des Sciences Pharmaceutiques, Université Paul Sabatier and Laboratoire de Chimie de Coordination, UPR CNRS 8241, 205 route de Narbonne, 31077 Toulouse cedex, France; pierre.verhaeghe@univ-tlse3.fr

Kinetoplastids are a group of flagellated parasites including *Leishmania* ssp. and *Trypanosoma* ssp. that are responsible for several neglected tropical diseases (NTD): visceral leishmaniasis (VL), human African trypanosomiasis (HAT) and Chagas disease (CD), globally responsible for about 30,000 annual deaths, according to the WHO. There are few drugs on the market against these parasitic diseases that affect people living in developing countries. Moreover, most of these drugs present severe side effects and are not orally available. In this worrying context, a new drug called Fexinidazole, a 5-nitroimidazole derivative developed by Sanofi and DNDi, was approved in 2018 by the EMA against HAT and is being evaluated in phase II against CD. Nevertheless, there is still no new chemical entity that clinically studied against VL. Working on nitroaromatic derivatives displaying anti-infective potential, our group identified two novel antileishmanial pharmacophores in 8-nitroquinolin-(1*H*)-one and 3-nitroimidazo [1, 2-*a*] pyridine series (Paloque, L., et al. *Eur. J. Med. Chem.* **2012**, *54*, 75–86; Castera-Ducros, C., et al. *Bioorg. Med. Chem.* **2013**, *21*, 7155–7164; Kieffer, C., et al. *Eur. J. Med. Chem.* **2015**, *92*, 282–294; Kieffer, C., et al. *Bioorg. Med. Chem.* **2015**, *23*, 2377–2386).

By synthesizing more than 200 derivatives, electrochemistry-guided pharmacomodulation studies progressively led to several potent and selective in vitro hit-compounds presenting IC_50_ values ranging 10 from 10 µM to nM against *L. donovani* (pro. & ama.), *L. infantum* (axe. ama.), *T. b. brucei* (trypo.) and *T cruzi* (epi.), low cytotoxicities on the human HepG2 cell line and high selectivity indices (from 10 to 400). We then demonstrated that, like for fexinidazole, these nitroaromatic molecules are selectively bioactivated by type 1 nitroreductases, probably leading to cytotoxic electrophilic reduction metabolites such as nitroso and hydroxylamine derivatives. In both series, hit-compounds were not genotoxic in the comet assay and even the mutagenicity Ames test was negative for several imidazopyridine derivatives. The determination of some preliminary in vitro pharmacokinetic parameters highlighted good microsomal stability (T_1/2_ > 40 min), high albumin binding (98%–99%) and blood–brain barrier diffusion (BBB PAMPA) in quinolinone series, whereas some metabolic issues needed additional work in imidazopyridine series to reach optimized hit-molecules that are now able to undergo in vivo evaluations on mouse models (Pedron, J., et al. *Eur. J. Med. Chem.* **2018**, *155*, 135–152; Pedron, J., et al. *ChemMedChem* **2018**, *13*, 2217–2228; Fersing, C., et al. *Eur. J. Med. Chem.* **2018**, *157*, 115–126; Fersing, C., et al. *ACS Med. Chem. Lett.* **2019**, *10*, 34–39).


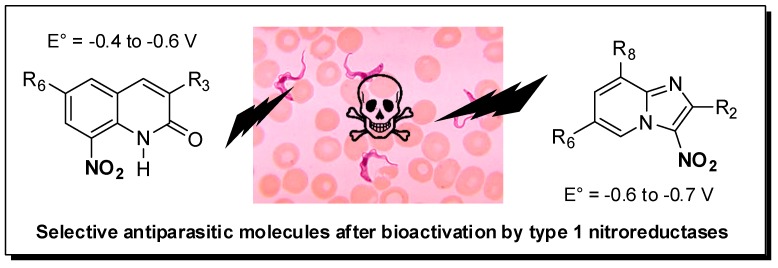


### 5.3. Small Molecule Drugs and Where to Find Them—Approaches to Hit Finding, New and Old (KL3)

WaringMichael J.Chemistry, School of Natural and Environmental Sciences, Newcastle University, Bedson Building, Newcastle upon Tyne NE1 7RU, UK; mike.waring@ncl.ac.uk

The technologies available to the medicinal chemist for finding hit molecules for proteins of interest have expanded rapidly in recent years. From extensive use of high-throughput screening to now well-established fragment-based methods to emerging technologies (Murray, C.W., Rees, D.C. *Nature Chem.* **2009**, *1*, 187–192) such as DNA-encoded chemical libraries (Favalli, N., et al. *FEBS Lett.* **2018**, *592*, 2168–2180), the range of complementary techniques is enabling more rapid drug-discovery, including approaches to more challenging targets. The central premises to all these approaches remain—how to select protein targets that are amenable to small molecule modulation in a manner compatible with ADMET properties and how to design screening libraries that generate the best quality hits for optimisation.

The talk will describe past optimisation programmes demonstrating where the hits came from and why these enabled the progression of the project. It will then outline how some of the identified concepts might be applied to future hit finding and some new approaches we are developing, such as Fraglite screening, which uses minimal halogenated fragments that express hydrogen bonding doublet motifs to assess druggability and inform future hit finding (Wood, D.J., et al. *J. Med. Chem.* **2019**, *62*, 3741–3752).

### 5.4. Development of Small Molecule Glial Cell Line-Derived Neurotrophic Factor (GDNF) Family Ligand Mimetics for the Treatment of Neurodegenerative Diseases and Pain (KL4)

SidorovaYulia A.MahatoArunSaarmaMartInstitute of Biotechnology, HiLIFE, University of Helsinki, FIN-00014 Helsinki, Finland; yulia.sidorova@helsinki.fi

Neurotrophic factors are secretory proteins that by binding to their cognate receptors trigger intracellular signalling promoting neuron survival, neurite outgrowth and regulate neuronal plasticity. Glial cell line-derived neurotrophic factor (GDNF) family consist of four factors GDNF, neurturin (NRTN), artemin (ARTN) and persephin (PSPN) that first bind to the GPI-anchored GDNF receptor family alpha (GFRa) and then activate the transmembrane tyrosine kinase RET. GDNF family ligands (GFLs) have neurotrophic effects on sensory, dopamine, sympathetic, parasympathetic, enteric neurons and motoneurons. A recently discovered novel member of the GFL family, a protein called GDF15, signals via the GFRAL-RET pathway and regulates body weight in many diseases (Saarma, M., Goldman, A. *Nature* **2017**, *550*, 195–197). GDNF and NRTN have been tested in phase II clinical trials on Parkinson′s disease patients showing modest effects that are in principle promising although statistically insignificant. ARTN has been tested in phase I clinical trials for neuropathic pain. To understand the molecular mechanisms of ligand receptor interactions, we solved the GDNF-GFRa1 complex structure at 2.35 Å resolution, identified amino acid residues involved in this interaction and residues possibly interacting with RET (Parkash, V., et al. *J. Biol. Chem.* **2008**, *283*, 35164–35172). Results of structural studies together with the notion that GFL proteins have poor pharmacological properties, encouraging us to develop small molecules that act similarly to GFLs. The major drawback hindering clinical use of GFLs is inability of these proteins to cross tissue barriers and limited tissue distribution, which restricts their clinical efficacy. We developed medium- and high-throughput methods to identify agonists of GFL receptors and screened a chemical library of 18,000 drug-like compounds. We identified a molecule named BT13 that activated GFL receptor RET and its down-stream signalling cascades in immortalized cells, as well as in sensory and dopamine neurons. In cultured dorsal root ganglion neurons BT13 activated Akt, Erk and Src intracellular signalling cascades and, in the ARTN-like fashion, stimulated elongation and arborisation of neurites of peptidergic neurons (Sidorova, Y.A., et al. *Front. Pharmacol.* **2017**, *8*, 365). In rodent spinal nerve ligation (SNL)-induced neuropathic pain model BT13, with equal efficacy with ARTN protein, the surgery-induced mechanical hypersensitivity and normalized the expression of multiple neuronal markers were decreased. BT13 penetrated through the blood–brain barrier, supported the survival of cultured dopamine neurons at 100 nM, alleviated motor symptoms of PD in a rat 6-OHDA model and showed a trend to increase the density of DA fibres in striatum. Using medicinal chemistry and different screening methods, we developed next generation GFL mimetics with improved efficacy, solubility and stability of the compounds. They are currently being tested in animal models of neuropathic pain and Parkinson′s disease. Novel GFL mimetics can be hopefully used also for therapeutic intervention in obesity and in palliative cancer care, as RET activation by GDF15 regulates body weight.

### 5.5. The Holy Grail of Opioid Analgesia? (KL5)

Cami-KobeciGerta^1^KoMei-Chuan^2^TraynorJohn R^3^HusbandsS M^1^1Department of Pharmacy and Pharmacology, University of Bath, Bath BA2 7AY, UK2Department of Physiology and Pharmacology, Wake Forest School of Medicine, Winston-Salem, NC, USA3Department of Pharmacology, University of Michigan, Ann Arbor, USA; s.m.husbands@bath.ac.uk

The opioid epidemic has rapidly evolved into a medical crisis, with recent figures suggesting 11.5 million people in the United States had misused prescription opioids and >17,000 deaths had been caused by commonly prescribed opioids in 2016 alone. A long sought-after but elusive solution has been the development of a strong but safe opioid analgesic—The Holy Grail of opioid research. Our approach has been to develop analgesics, such as BU08028 (Ding, H., et al. *PNAS*, **2016**, *113*, E5511–E5518), with partial agonist activity at both mu and nociceptin/orphanin FQ peptide receptors, as evidence suggests a synergistic interaction between these receptors for analgesia. BU08028 proved to be a potent, long-acting analgesic in primates and did not cause respiratory dependence or reduce respiration. Having defined a pharmacophore for mixed mu and nociception receptor binding, we were able to design a further series of mixed ligands based on the oxymorphone scaffold. BU10038 was identified from within this series as having an almost identical receptor profile to BU08028 and has now been evaluated in a battery of assays to define its analgesic and side effect profile (Kiguchi, N., et al. *Brit. J. Anaesthesia*, **2019**, *122*, e146–e156). Following systemic administration in non-human primates, BU10038 (0.001–0.01 mg kg^−1^) dose-dependently produced long-lasting antinociceptive and antihypersensitive effects. Unlike the standard analgesic oxycodone, BU10038 lacked reinforcing effects (i.e., had little or no abuse liability), and compromised the physiological functions of primates including respiration, cardiovascular activities and body temperature at antinociceptive doses and at a 10–30-fold higher dose (0.01–0.1 mg kg^−1^). Unlike morphine, BU10038 did not cause the development of physical dependence and tolerance after repeated and chronic administration. This work strongly supports the development of bifunctional MOP/NOP agonists as improved analgesics and an alternative solution for the ongoing prescription opioid crisis.


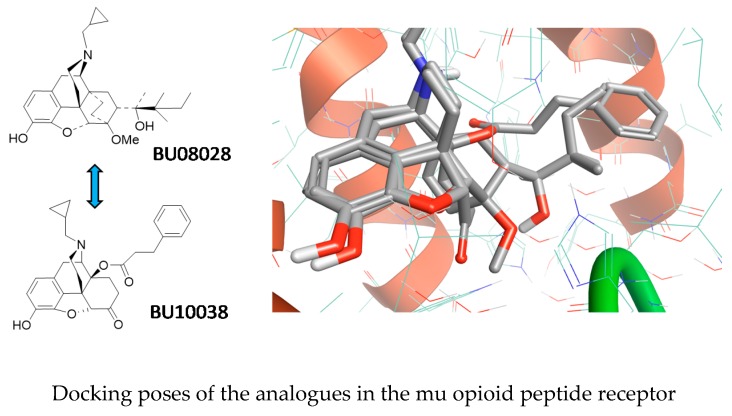


Funding from the National Institute on Drug Abuse and Orexigen Therapeutics.

### 5.6. Design of Matrix Metalloproteinase Inhibitors for Inflammatory Bowel Disease (KL6)

PigottMariaWangJunO′SullivanShaneMedinaCarlosGilmerJohn FSchool of Pharmacy and Pharmaceutical Sciences, Trinity College Dublin, D2, Ireland; gilmerjf@tcd.ie

Matrix metalloproteinases (MMPs) is an umbrella term for a large family of widely distributed enzymes that influence diverse biochemical processes relevant to normal physiology and to disease. MMPs are interesting drug targets but they are challenging because of their distribution, range of effects and similarity. The so-called gelatinase B enzyme, also known as MMP-9, is a key player in the progression of inflammatory bowel disease (IBD) and a marker of inflammation and disease progression (O′Sullivan, S., et al. *Mediat. Inflamm.* **2015**, *2015*, 1–18). This talk will describe the biology of MMP-9 in IBD and provide an overview of our attempts to design inhibitors that selectively affect its function. These efforts relate to three broad strategies: (i) attempts at increasing MMP-9 selectivity in the pyrimidinetrione class through conventional medicinal chemistry approaches (Wang, J., et al. *Bioorg. Med. Chem*. **2011**, *19*, 16, 4985–4999); (ii) hybrid tactics using nitric oxide mimetic functionality to influence MMP transcription (O′Sullivan, S., et al. *Br. J. Pharmacol*. **2017**, *174*, 512–524; Wang. J.; O′Sullivan, S., et al. *J. Med. Chem*. **2012**, *55*, 2154–2162); (iii) quasi pharmaceutical strategies to confine broadly unselective pyrimidinetrione inhibitors to disease tissue through manipulation of chemical properties. The outcomes of these attempts and lessons learned will be related through data from in vitro/in vivo models of ulcerative colitis.

### 5.7. Computational Medicinal Chemistry Approaches for GPCR Structure-Based Drug Discovery (KL7)

De GraafChrisSosei Heptares; Chris.DeGraaf@heptares.com

Novel crystal structures of GPCR-ligand complexes solved at Sosei Heptares and elsewhere continue to reveal a diversity of potential ligand binding sites. Emerging sets of GPCR crystal structures of multiple diverse ligands bound to closely related receptors furthermore finally enable a protein structure-based view of how different ligands bind this major drug target class.

This presentation will address several important repercussions and learnings from the analysis of GPCR structures for ligand design that should be transferable and relevant for many targets, both GPCRs and enzymes, including:

- Caveats in using pharmacophore-based similarity principles for modeling receptor ligand complexes different ligand chemotypes.
- The important roles of lipophilic hot spots and water networks as drivers of GPCR druggability, ligand binding and selectivity.
- Binding mode diversity of (chemically similar) ligands across the structural GPCRome.

This presentation will show how the breakthroughs in GPCR structural biology can be complemented by computational and experimental studies for a more accurate description and prediction of molecular and structural determinants of ligand-receptor binding affinity, kinetics, potency and selectivity. Integrated cheminformatics workflows will be described that combine structural, pharmacological and chemical data to explore receptor–ligand interaction space and steer structure-based virtual ligand screening. Novel cheminformatics-driven molecule design approaches will be discussed, combining retrosynthetic analysis, library enumeration approaches (e.g., matched molecular pairs analysis) and recurrent neural network-driven molecule generation.

We will discuss how the accumulated GPCR structural chemogenomics data can be used to construct customized structure-based medicinal chemistry toolboxes for hit optimization and library design. Orthogonal physics-based (Molecular Dynamics, e.g., Free Energy Perturbation FEP+, WaterMap from Schrödinger) and empirical (e.g., GRID and WaterFLAP from Molecular Discovery) structure-based drug design methods will be presented to target lipophilic hotspots, water networks and cryptic ligand binding pockets for a variety of GPCR subfamilies.

### 5.8. Phenotypic Activity Driven Target Identification and Discovery of Drug Candidates (KL8)

WyattPaulDrug Discovery Unit, Wellcome Centre for Anti-Infectives Research, University of Dundee, School of Life Sciences, Dundee DD1 5EH, UK; P.G.Wyatt@dundee.ac.uk

There are considerable opportunities for drug discovery centres based in universities to generate benefit to patients, provided they do not compete with or merely replicate BioPharma. The centres have to develop activities complementary to, or in partnership with the BioPharma industry. These include partially de-risking novel drug discovery targets, or tackling neglected and orphan diseases. The Drug Discovery Unit seeks to achieve this by combining a globally rare university-based, fully integrated drug discovery group capable of delivering new drug candidates with a world-class life sciences research environment and multiple partners to develop the drug discovery approaches to major global diseases.

The talk describes how we are delivering much needed clinical candidates, for example, malaria and visceral leishmaniasis (two) by taking a phenotypic approach to hit identification and optimisation, using mode of action studies to identify novel drug targets and to manage a portfolio of phenotypically derived projects.

### 5.9. Resin Acid-Driven Innovations in Antimicrobial Research (KL9)

MoreiraVânia M.^1^,^2^1Strathclyde Institute of Pharmacy and Biomedical Sciences, University of Strathclyde, United Kingdom, 161 Cathedral Street, G4 0RE Glasgow; vania.moreira@strath.ac.uk2Drug Research Program, Division of Pharmaceutical Chemistry and Technology, Faculty of Pharmacy, University of Helsinki, Finland, Viikinkaari 5 E, P.O. Box 56, FI-00014 University of Helsinki

Humans have employed resins for esthetic, ceremonial or therapeutic uses for millennia (Klemens, F., Dieter, G. “Resins, Natural”. Ullmann′s Encyclopedia of Industrial Chemistry, **2000**). Gum rosin, in particular, has an annual worldwide production of more than 1 million tons and is used as an ingredient for inks, varnishes, adhesives, cosmetics, medicines and chewing gums. The ecological role of resins is protective, that is, when trees are cut, the volatiles in the resin evaporate leaving the solid portion that contains “resin acids”, which shelter them from external stress and invaders, promoting healing.

Our research has revealed that these “resin acids”, belonging to the class of abietane-type diterpenoids, have antimicrobial and anti-biofilm activity towards gram-positive bacteria and can be exploited for human use (Fallarero, A., et al. *Int. J. Mol. Sci*. **2013**, *14*, 12054–12072). Through chemistry, we have created a library of over 50 new chemically pure “resin acids” derivatives, which includes, to the best of our knowledge, the most potent agents reported within their class so far (Manner, S., et al. *Eur. J. Med. Chem.* **2015**, *102*, 68–79; Moreira, V.M., et al. WO 2016/051013 A1, 7th April **2016**). It is noteworthy that this effect is accompanied by a good tolerability profile and low potential for spreading resistance. Moreover, combination of these compounds with nanocellulose, an advanced biopolymer with excellent mechanical properties and biocompatibility, resulted in innovative and cost-effective functional surfaces, bearing a broad spectrum of action against bacteria, high biocompatibility and no cross-resistance with drug-resistant strains (Hassan, G., et al. *ACS Sustainable Chem. Eng.* **2019**, *7*, 5002–5009). This presentation will detail the outcomes of our work with this class of compounds and further illustrate how natural products and their derivatives foster innovation in antimicrobial research.

Acknowledgements: The Finnish Funding Agency for Technology and Innovation (project 1297/31/2016).

### 5.10. Glycoconjugates Disrupting Bacterial and Fungal Adhesion Mechanisms (KL10)

GouinSébastien G.University of Nantes, CEISAM, Chimie Et Interdisciplinarité, Synthèse, Analyse, Modélisation, UMR CNRS 6230, UFR des Sciences et des Techniques, 2, rue de la Houssinière, BP 92208, 44322 NANTES Cedex 3, France; sebastien.gouin@univ-nantes.fr

Increasing resistance to antibiotics or antifungals is a serious health problem, which is worsening with the constant identification of strains resilient to commonly available chemotherapeutic agents. Among the therapeutic alternatives developed at the academic level, the anti-adhesive strategy has seen a growing interest in the last 25 years (Cecioni, S., et al. *Chem. Rev.* **2015**, *115*, 525–561). The concept is to disrupt the lectin-mediated adhesion of the pathogen to eukaryotic cells. This therapeutic approach should be less prone to the development of resistances and selection pressures, as the pathogens are not killed during the decolonisation process.

**Figure 1:**
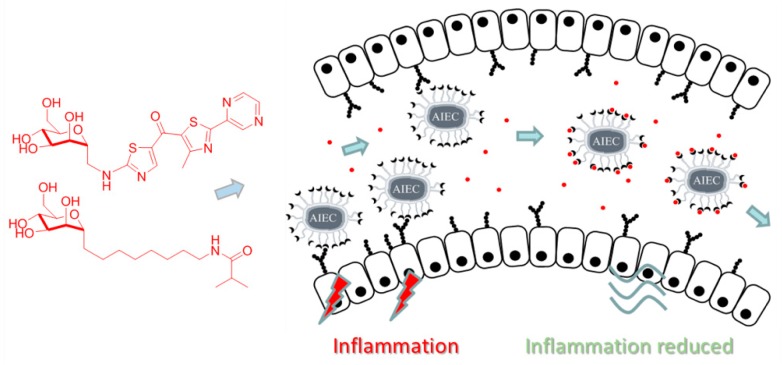
Illustration of the antiadhesive concept. Oral administration of *Escherichia coli* antiadhesives in Crohn′s disease mouse model was shown to lower the bacterial load in the gut and to reduce the inflammatory syndromes. AIEC: adherent-invasive *E. coli*.

Our group is interested in developping antagonists of carbohydrate-binding and processing proteins (adhesins and glycosidases) to disrupt the attachment of pathogenic bacteria and fungi to host cells. We will discuss the design of mono- and multivalent glycoconjugates and their in vitro and in vivo antiadhesif potential. A specific focus will be made on antagonists of adhesins (Figure 1) (Brument, S., et al. *J. Med. Chem.* **2013**, *56*, 5395–5406; Brissonnet, Y., et al. *J. Am. Chem. Soc.* **2013**, *135*, 18427–18435; Brissonnet, Y., et al. *Chem. Eur. J.* **2019**, *25*, 2358–2365; Alvarez Dorta, D., et al. *ChemBioChem* **2016**, *17*, 936–952) or invasive aspergillosis (Lehot, V., et al. *Chem. Eur. J.* **2018**, *24*, 19243–19249).

### 5.11. Drug-Like Properties and Ligand Efficiency-Guided Optimisation (KL11)

LeesonPaulPaul Leeson Consulting Ltd. and School of Pharmacy, University of Nottingham, UK; leesonpd@gmail.com

It has been acknowledged that significant attrition and delay in clinical drug development pipelines has been caused by poor compound properties such as inadequate pharmacokinetics and poor solubility. Generic compound quality guidelines, such as the rule of five, may not always be helpful because differing drug binding sites on biological targets can strongly influence the allowable physicochemical property ranges of their ligands. The application of ligand efficiency measures, which quantify in vitro activities per unit of a physical property such as size or lipophilicity, provides an improved perspective of compound quality over physicochemical properties alone. Thus, candidate drugs and approved drugs are frequently amongst the most ligand efficient for their specific target, on the basis of measures using both size (ligand efficiency, LE) and lipophilicity (ligand lipophilic efficiency, LLE). This is seen even with drugs having extreme physicochemical properties, such as those breaking the rule of five, for example, the new raft of drugs for treatment of the hepatitis C virus. The desirable optimisation destination in the property efficiency space of a novel target can be anticipated once enough screening data are available, usually in the hit-to-lead phase. In conjunction with the application of multiparameter ADMET optimisation, the approach can help to refine drug design options, prioritise synthesis and reduce the numbers of compounds made (Young, R.J., et al. *J. Med. Chem.* **2018**, *61*, 6421–6467).

### 5.12. Natural Products and Analogues as Potential Therapeutic Agents (KL12)

CortesDiego^1^SanzMaría Jesús^1^,^2^CabedoNuria^2^1Department of Pharmacology, University of Valencia, Burjassot, Spain, 461002Biomedical Research Institute INCLIVA, 46010 Valencia, Spain; ncabedo@uv.es

**Figure 2:**
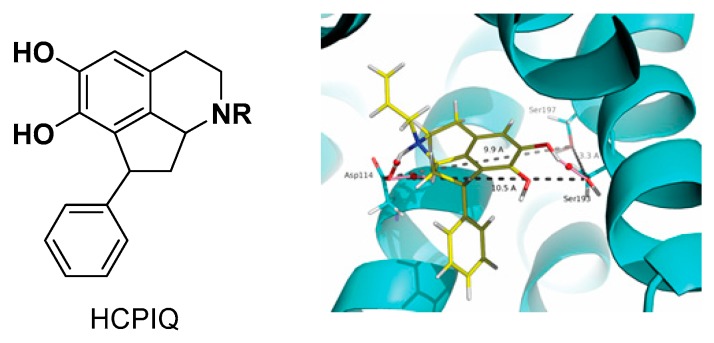
Molecular graph of the molecular interactions between hexahydrocyclopenta-IQ (HCPIQ) 3e and hydrophobic residues: Val115, Ile183, Ile184, Trp386, Phe389, Phe390, His393, Thr412 and Tyr416 of D_2_ dopamine receptors (DR).

Natural products have been the most valuable inspiration for the synthesis of novel therapeutic agents. Among them, heterocyclic moieties are prevalent in pharmacologically relevant drugs (Cortes, D. Bioactive Secondary Metabolites: The Drugs that Nature provides. **2018**. Cortes D. (Ed). ISBN-10: 171877768X; ISBN-13: 978–1718777682. CreateSpace). In the course of our research on synthesis of bioactive heterocycles, we synthetized novel isoquinolines as dopaminergic ligands, including tetrahydroisoquinolines carbamates (Parravicini, O., et al. *J. Mol. Model.* **2017**, *23*, 273), hexahydrocyclopenta-IQ (HCPIQ)(Figure 2) (Parraga, J., et al. *Eur. J. Med. Chem*. **2016**, *122*, 27–42), aporphines (Moreno, L., et al. *Bioorg. Med. Chem. Lett.* **2013**, *23*, 4824–4827), phenanthrenes (Moreno, L, et al. *Bioorg. Med. Chem. Lett.* **2013**, *23*, 4824–4827) and protoberberines (Párraga, J., et al. *Eur. J. Med. Chem*. **2013**, *68*, 150–166). Dopaminergic ligands can bind to subfamily D_1_-like (D_1_, D_5_) and D_2_-like (D_2_-D_4_) dopamine receptors (DR) to “restore” the dopaminergic pathway. From a therapeutic point of view, DR are the main target for antipsychotic (neuroleptics) and antiparkinsonian drugs. Recently, we demonstrated by binding experiments and functional assays that some of these compounds displayed affinity for DR with high selectivity for D_2_-like DR, behaving as dopaminergic agonists. Molecular modelling studies have established the main molecular interactions that stabilize the different ligand-receptor complexes (Parravicini, O., et al. *J. Mol. Model.* **2017**, *23*, 273; Parraga, J., et al. *Eur. J. Med. Chem*. **2016**, *122*, 27–42).

In recent years, considerable attention has been paid on melatonergic (MT) receptor agonists given their application for treating insomnia and depression. We synthetized novel hexahydroindenopyridines (HHIP), which were able to bind MT1 and/or MT2 receptors. These HHIP emerged as promising leads for the development of new melatoninergic agents (Parraga, J., et al. *Eur. J. Med. Chem*. **2014**, *30*, 700–709).

This work was funded by grants SAF2017–89714-R, SAF2014–57845R, CP15/00150 and PI18/01450 from Ministerio de Ciencia, Innovación, Instituto de Salud Carlos III and co-funded by European Union (ERDF/ ESF) “Investing in your future”.

## 6. Young Researcher Communications

### 6.1. Pyrroloquinolones as Novel Dual-Targeting and Dual-Stage Oral Efficacious Antimalarials (YRC01)

da SilvaGustavo^1^,^2^CoelhoLis^3^FontinhaDiana^4^SilvaVera L. M.^2^SilvaArtur M. S.^2^MoreiraRui^1^1iMed.ULisboa, Faculdade de Farmácia da Universidade de Lisboa, Lisboa, Portugal, 1649–0032Department QOPNA, Departamento de Química da Universidade de Aveiro, 3810–193 Aveiro, Portugal3Centro da Malária e Doenças Tropicais, IHMT, Universidade Nova de Lisboa, 1349–008 Lisboa, Portugal4Instituto de Medicina Molecular, Faculdade de Medicina Universidade de Lisboa, 1649–028 Lisboa, Portugal; galfdsilva@ff.ulisboa.pt

**Figure 3:**
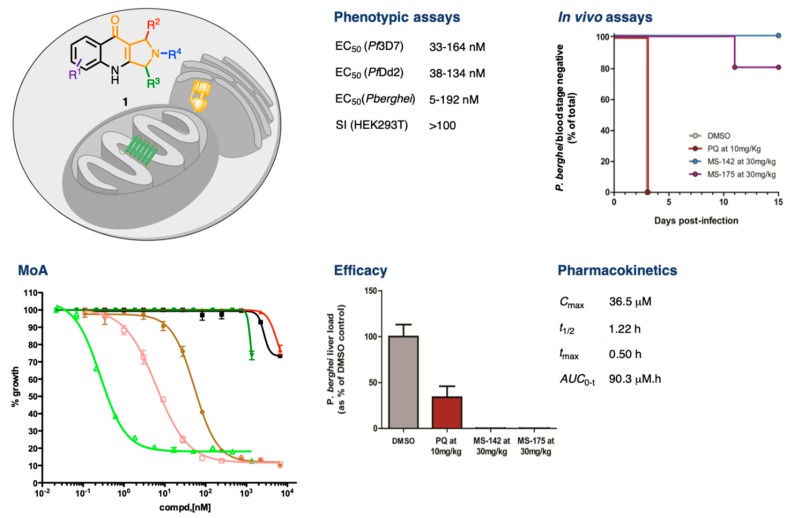
Structure and relevant results of our lead compounds.

In this communication, we report the results from a medicinal chemistry programme focused on a pyrroloquinolone (PQ) series targeting the *Plasmodium falciparum* mETC (mitochondrial electron transport chain). This previously unknown series **1** was tested against blood- and liver-stage malaria parasites and our hit compound revealed excellent dual-stage inhibitory activity. The plausible structure–activity relationships and the inhibition of mETC components (cytochrome *bc*_1_, dihydroorotate dehydrogenase) will be discussed in this communication. In addition, our hit compound interfered with calcium homeostasis in transgenic malaria parasites. The interactions between the lead compounds and target were confirmed by docking. Our hit compound also revealed a good selectivity index, in vivo efficacy and favourable pharmacokinetic properties (Figure 3).

This work was supported by Fundação para a Ciência e Tecnologia (FCT), FEDER and PT2020 through projects UID/DTP/04138/2013 and UID/QUI/00062/2013. We also thank FCT for the fellowships SFRH/BPD/108807/2015 (V.L.M.S.) and SFRH/BD/103412/2014 (G.S.).

### 6.2. Molecular Docking, Semisynthesis and Biological Evaluation of Garcinoic Acid Amides as 5-Lipoxygenase Inhibitors (YRC02)

DinhChau Phi^1^VilleaAlexiaBarbéAnais^1^ToThuy Linh^1^NeukirchKonstantin^2^ViaultGuillaume^1^TemmlVeronika^3^KoeberleAndreas^2^SchusterDaniela^4^WerzOliver^2^RichommePascal^1^HelesbeuxJean-Jacques^1^SéraphinDenis^1^1SONAS, EA921, UNIV Angers, SFR QUASAV, Faculty of Health Sciences, Department of Pharmacy, 16 bd Daviers, 49045 Angers Cedex 01, France2Chair of Pharmaceutical/Medicinal Chemistry, Institute of Pharmacy, Friedrich-Schiller-University Jena, 07743 Jena, Germany3Institute of Pharmacy/Pharmacognosy University of Innsbruck, 6020 Innsbruck, Austria4Department of Pharmaceutical and Medicinal Chemistry, Paracelsus Medical University Salzburg, 5020 Salzburg, Austria; chauphi.dinh@univ-angers.fr

Prostaglandins and leukotriens (LTs), produced from arachidonic acid by cyclo-oxygenase and 5-lipoxygenase (5-LOX) pathways, respectively, are key mediators in inflammation (Funk, C. D. *Science* **2001**, *294*, 1871–1875). Our recent results showed an anti-inflammatory activity of ω-oxidized tocotrienols by inhibiting 5-LOX (Pein, H., et al. *Nat. Commun.* **2018**, *9*, 3834). Amongst these compounds, δ-garcinoic acid (δ-GA) was the most active one. It inhibited LTs formation in 5-LOX and polymorphonuclear leukocyte assays with IC_50_ at 0.04 ± 0.02 µM and 0.35 ± 0.08 µM, respectively. Molecular docking with an in-house dataset was performed and identified new allosteric binding site for these compounds.


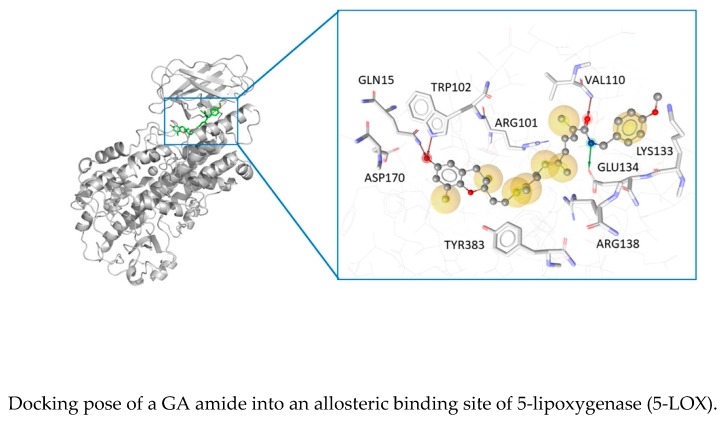


In order to further understand the mode of action and to improve the 5-LOX inhibitory activity of δ-GA or its pharmacological profile, the rational design of a series of amides followed by their semisynthesis was carried out. Here, we present a dataset of novel δ-GA-based anti-inflammatory agents with promising in vitro and in cellulo anti-inflammatory activity.

### 6.3. Polypharmacological Compounds in Alzheimer′s Disease: Innovative 5-HT_4_R Agonist with Promising in Cellulo Antioxidant Properties (YRC03)

LanthierCaroline^1^liparuloIrene^2^PayanHugo^3^LecouteyCédric^1^SinceMarc^1^DavisAudrey^1^ClaeysenSylvie^3^BolognesiMaria-Laura^2^DallemagnePatrick^1^RochaisChristophe^1^1Normandie Univ, UNICAEN, CERMN, 14000 Caen, France2Department of Pharmaceutical Sciences, University of Bologna, Via Belmeloro 6, 40126 Bologna, Italy3IGF, Univ. Montpellier, CNRS, INSERM, Montpellier, France; caroline.lanthier@gmail.com

In a world where life expectancy is increasing, Alzheimer disease (AD) is the main cause of dementia, and affects approximatively 17% of people who are older than 75 years in France. This is a progressive neurodegenerative disorder characterized by memory loss and cognitive decline. Despite the fact that the physiopathology of AD is not entirely known at the current time, some molecular causes were found such as the ß-amyloid peptides aggregation, tau-dependent neurofibrillary tangles, as well as oxidative stress and neuroinflammation. Currently, treatments available for patients are mainly acetylcholine esterase (AChE) inhibitors, which only have symptomatic benefits and do not cure AD. Thus, there is still a strong medical need in the AD population.

**Figure 4:**
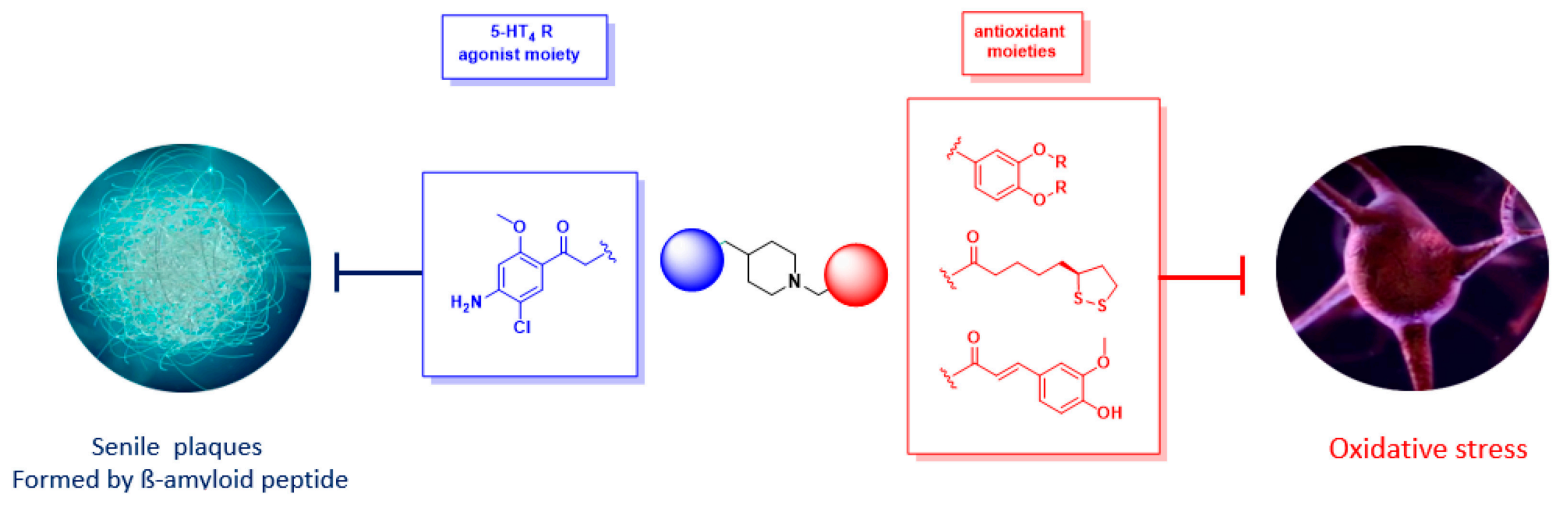
Targeted structure, with 5-HT4R agonist moiety in blue and antioxidant moieties in red.

In this context, the concept of multi-target directed ligands (MTDLs) was applied to design a drug with several therapeutic targets. The envisaged MTDL (targeted structure—Figure 4) should be able to limit the development of ß-amyloid plaques obtained by the aggregation of ß-amyloid peptides (Aß). Indeed, our compounds are designed to promote the cleavage of amyloid protein precursor (APP) by α -secretase activation in order to produce a neuroprotective and soluble peptide sAPPα. This is the role of the 5HT4R agonists, which are already studied in the CERMN (Centre d’Etudes et de Recherche sur le Médicament de Normandie) in other MTDL projects and led to the discovery of donecopride (blue part—Figure 1) (Rochais, C., et al. *J. Med. Chem*., **2015**, 58, 3172–3187). On the other hand, it appears that oxidative stress plays a central role in AD (Rosini, M., et al. *J. Med. Chem*., **2014**, 57, 2821–2831). Adding antioxidant moiety such as polyphenol, lipoic and ferulic acid (red part—Figure 1) could trap free radicals or reactive oxygen species (ROS) and also have a neuroprotective effect. This aspect has been widely studied in Prof. Maria-Laura Bolognesi′s laboratory over the years (Bolognesi, M.-L., et al. *J. Med. Chem*., **2009**, 52, 7883–7886). To that end, different compounds will be designed and synthesized, with both the expertise of CERMN and Prof. Maria-Laura Bolognesi, in order to evaluate their in vitro/in vivo properties regarding their agonist activity on 5-HT_4_R and antioxidant properties. The development and promising in vitro/in cellulo results of the chloroaniline′s moiety line will be described in this presentation.

### 6.4. Reactivity of Pazopanib and Sunitinib Aldehyde Metabolites toward Proteins: Potential Involvement in Hepatotoxicity and Drug–Drug Interactions (YRC04)

PaludettoMarie-Noëlle^1^StiglianiJean-Luc^2^RobertAnne^2^Bernardes-GénissonVania^2^ChatelutEtienne^1^PuissetFlorent^1^ArellanoCécile^1^1Centre de Recherches en Cancérologie de Toulouse (CRCT), University of Toulouse, 31037 Toulouse, France2Laboratoire de Chimie de Coordination du CNRS (LCC-CNRS), University of Toulouse, 31077 Toulouse, France; paludetto.marienoelle@iuct-oncopole.fr

Pazopanib and sunitinib are tyrosine kinase inhibitors (TKI) approved for the treatment of various cancers. Their hepatotoxicity has been highlighted by the black boxed warnings issued by the FDA in their drug labels. These TKI are mainly metabolized by hepatic cytochromes P450 (CYP), and previous in vitro studies have suggested that they are CYP3A4 time-dependant inhibitors (Kenny, J. R., et al. *Pharm. Res.* **2012**, *29*, 1960–1976; Filppula, A. M., et al. *Drug Metab. Dispos*. **2014**, *42*, 1202–1209). New reactive metabolites (RM) with aldehyde structures have been discovered during pazopanib and sunitinib metabolism (Paludetto, M-N., et al. *J. Med. Chem*. **2018**, *61*, 7849–7860). Aldehydes are hard electrophiles that have a high reactivity potential toward proteins and that are thought to be responsible for CYP inactivation, drug–drug interactions (DDI) and hepatotoxicity (Kalgutkar, A. S. *Chem. Res. Toxicol*. **2017**, *30*, 220–238).

The reactivity of pazopanib and sunitinib aldehyde RM toward different hepatic (human liver microsomes (HLM), recombinant CYP3A4) and plasma (albumin) proteins was investigated by LC-MS/MS to better understand how these RM could be involved in liver toxicity and DDI. Because of the presence of a free amino group on its side chain, lysine could be a good target for reactive electrophiles. Reactions between aldehydes and amines affording imine derivatives are known to be reversible in aqueous media. Thus, adduct formation with these different proteins was examined after reductive amination of imine derivatives by NaBH_3_CN in vitro and after proteolytic digestion. Preliminary studies were carried out with free lysine and aldehyde RM generated beforehand by biomimetic oxidation reactions catalysed by metalloporphyrins. Lysine adduct formation was then studied with aldehyde RM generated in situ in HLM and recombinant CYP3A4 incubations. Aldehyde-lysine adducts on both CYP3A4 and plasma proteins were detected for the first time by LC-MS for pazopanib and sunitinib and characterized by the related [M+H]^+^ ions and MS/MS fragmentation patterns. In addition, the formation of aldehyde RM in vivo was investigated in plasma samples from patients treated, separately, with pazopanib or sunitinib. These aldehyde RM have been unambiguously detected in patient plasma samples during drug monitoring. Thus, it could be hypothesized that the coupling of pazopanib or sunitinib aldehydes with lysine residues of CYP3A4 and hepatic proteins may be involved both in hepatotoxicity and DDI.

### 6.5. Click Chemistry Tools to Unravel New Targets in Malaria (YRC05)

GriloJorge^1^,^2^FidalgoLara^1^FontinhaDiana^2^AnacletoBeatriz^1^SaundersCharlie^3^TateEdward W.^3^MoreiraRui^1^PrudêncioMiguel^2^RessurreiçãoAna S.^1^1Research Institute for Medicines—iMed. ULisboa, Faculdade de Farmácia da Universidade de Lisboa, 1649–003 Lisbon, Portugal2iMM—Instituto de Medicina Molecular, Faculdade de Medicina da Universidade de Lisboa, 1649–028 Lisbon, Portugal3Department of Chemistry, Imperial College London White City Campus, London W12 0BZ, UK; jhfgrilo@ff.ulisboa.pt

Despite the best efforts and investment from the last decade, malaria′s death toll still amounts to nearly half a million people a year, and no reduction of the diagnosed cases was observed in the last two years. Furthermore, parasite resistance is already reported for every drug class in clinical use. These makes this protozoan infection a major public health concern mainly in the tropical regions of the globe (WHO. *World Malaria Report*. **2018**). To overcome these hurdles, it is critical to advance new drugs with different mechanisms of action.

With that in mind, we have already disclosed Torin2, known as an ATP-competitive mTOR kinase inhibitor (Liu, Q., et al. *J. Med. Chem*. **2011**, *54*, 1473), as a portent antimalarial lead compound that possesses in vivo activity in mice against the blood and liver stages. Compound activity was shown to be independent of the host mTOR pathway and does not involve the mechanisms already knows for drugs in clinic (Hanson, K. K., et al. *Proc. Natl. Acad. Sci. U.S.A.* **2013**, *110*, E2838).

**Scheme 1:**
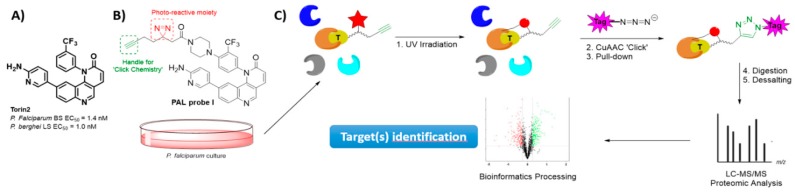
(**A**) Torin2 hit compound; (**B**) structure of the photo-affinity labelling probe I, derived from Torin2 by the introduction of a diazirine photoreactive moiety (in red) and a terminal alkyne handle for CuAAC chemistry (in green) and (**C**) schematic overview of the methodology applied for *Plasmodium falciparum* cell-based proteome profiling. In red: Diazirine before (star) and after (circle) irradiation. In pink: TAMRA and/or Biotin azide capture reagents.

Here, we report the development of Torin2 photoaffinity-based probes to deconvolute the molecular target(s) of this compound in the parasite. This was achieved through the modification of the core scaffold with a photocrosslinker and a handle suitable for ‘click chemistry’. Furthermore, we disclose the application of these chemical tools in a mass spectrometry-based proteome profiling of *P. falciparum* lysates and live cultures of the disease blood stage (Scheme 1). The results thus obtained are fundamental optimization of Torin2-based compounds as a new alternative to current antimalarials, a key breakthrough to address the existing therapeutic gap.

Acknowledgements: FCT-Portugal, (PTDC/QEQ-MED/7097/2014)(PD/BD/128260/2016)(IF/01034/2014).

### 6.6. The Development of a 6-Chloro-3-(Thiazole)Methyl-Quinazolin-4(3H)-One Series as Inhibitors of PqsR, the Transcriptional Regulator of the Pqs Quorum Sensing System in Pseudomonas aeruginosa. (YRC06)

GrossmanScott^1^SoukariehFadi^2^WilliamsPaul^2^CámaraMiguel^2^StocksMichael J.^1^1School of Pharmacy, Nottingham NG7 2RD, UK2School of Life Sciences, National Biofilms Innovation Centre, Centre for Biomolecular Sciences, University of Nottingham, Nottingham, Nottinghamshire NG7 2RD, UK; scott.grossman@nottingham.ac.uk

As the rise in antimicrobial resistance continues to reduce treatment options for a variety of pathogens at an alarming rate, the need for alternate antimicrobial agents grows ever more urgent. One such approach gaining ever more interest is the attenuation of virulence through the silencing of quorum sensing, a form of community-based cell-to-cell communication. The opportunistic pathogen *Pseudomonas aeruginosa* is the greatest contributor to morbidity in cystic fibrosis patients, and causes chronic and persistent infections in immunocompromised patients, particularly in a hospital environment.

Previous studies have evidenced the attenuation of virulence through the suppression of its alkylquinolone-based quorum sensing system, *Pseudomonas* quinolone system (*pqs*) (Ilangovan, A., et al. *PLoS Pathog.* **2013**, *9*, e1003508). Herein, we report the synthesis and biological evaluation of a novel series of 6-chloro-3-(thiazole)methyl-quinazolin-4(*3H*)-one compounds (Figure 1) through the determination of their biological activity using both *P. aeruginosa* PAO1-L mCTX:P*_pqsA_*-*lux* and PA14 mCTX:P*_pqsA_*-*lux* bioreporter strains. We report structural evaluation of their binding mode through generating protein–ligand crystal structures of this class of novel inhibitors in the PqsR ligand binding domain. The structure–activity relationship (SAR) study verified the binding mode to be comparable with that of the natural agonists HHQ and PQS, whilst providing lead *pqs* inhibitors with IC_50_s in the 100 nM range.


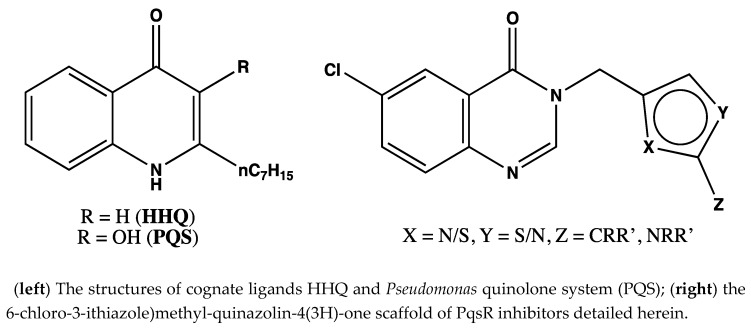


## 7. Posters

### 7.1. Design and Synthesis of New Integrated Stress Response Inhibitor (ISRIB) Analogues Targeting eIF2B as Potential Candidates for Enhancing Protein Synthesis Rate (P01)

AlardAkeel Abo^1^,^2^RonDavid^3^Crespillo-CresadoAna^3^ZyryanovaAlisa^3^FromontChristophe^1^BarrettDave^1^OteriCatherine^1^GershkovichPavel^1^LeeJong Bong^1^KellamBarrie^1^FischerPeter^1^1Center for Biomolecular Science, University of Nottingham, Nottingham NG7 2RD, UK2Department of Pharmaceutical Chemistry, Faculty of Pharmacy, University of Kufa, Najaf 54001, Iraq3Cambridge Institute for Medical Research, University of Cambridge, Cambridge CB2 0XY, UK; paxaa30@nottingham.ac.uk

**Figure 5:**
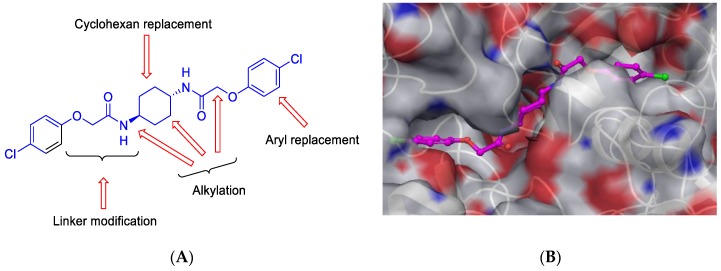
(**A**) General overview of integrated stress response inhibitor (ISRIB) structural modification investigated in this study, (**B**) binding mode of ISRIB in the active site of eIF2B.

Neurodegenerative diseases represent the major cause of dementia, which is predominantly a disease of the elderly, and is characterised by a decline in cognitive functions and memory. All these diseases share a similar aetiology, that is, the production and accumulation of misfolded proteins in the brain. This results in increasing the activity of the protein kinase RNA-like endoplasmic reticulum kinase PERK/eIF2α-P pathway of the unfolded protein response (UPR), which in turn leads to the inhibition of protein synthesis. According to a recent study the small-molecule integrated stress response inhibitor (ISRIB) reduces the level of eIF2α-P by activating eukaryotic initiation factor 2B (eIF2B), a guanine nucleotide exchange factor (GEF) for eIF2α, resulting in enhancement of protein synthesis based on in vivo study in the cell-based luciferase reporter assay (Sidrauski, C., et al. *Elife.* **2013**, *2*, e00498; Halliday, M., et al. *Cell death dis.* **2015**, *6* (3), e1672.). Herein, ISRIB analogues were designed and synthesised, focusing on modifications of the central core cyclohexyl ring, *O*-arylglycolamide side chains and terminal aromatic rings of the prototype ISRIB molecule (Figure 5). Synthesis of truncated ISRIB analogues revealed that even subtle modifications have an obvious impact on activity. Central core ring was also explored, and it was found that its replacement with 1,4-diaminopiperazine and 1,4-diaminopiperdine retained high activity. Furthermore, to assist in observe ISRIB′s interaction with eIF2B, we designed and synthesised fluorescently label derivative, considering the most active compound, with a modified aryl ether portion that was then used to elaborate the fluorescently label compound. The derivatized compound contained a flexible linker terminating in a 5/6-carboxyfluorescein moiety (Zyryanova, A. F., et al. *Science* **2018**, *359* (6383), 1533–1536).

### 7.2. Novel Synthesis of 3-Fluoro and 3,3-Difluoro Substituted β-Lactams: Evaluation as Potential Anticancer Agents (P02)

MalebariAzizah^1^,^2^MeeganMary J.^2^1Department of Pharmaceutical Chemistry, College of Pharmacy, King Abdulaziz University, Jeddah, KSA2School of Pharmacy and Pharmaceutical Sciences, Trinity Biomedical Sciences Institute, 152–160 Pearse Street, Trinity College Dublin, Dublin 2, Ireland; melibaa@tcd.ie

**Scheme 2:**
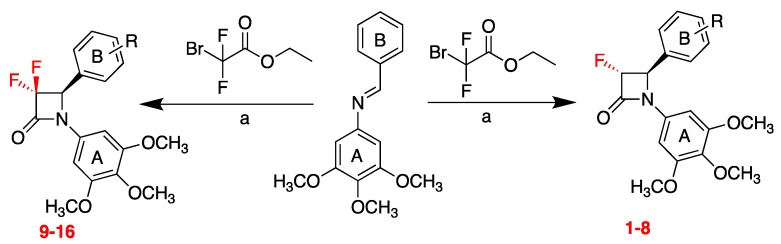
Synthesis of 3-fluoro and 3,3-difluoro β-lactams by the microwave assisted Reformatsky reaction. Reagents and conditions: (**a**) Zn dust, (CH_3_)_3_SiCl, 40 °C, 2 min, microwave; C_6_H_6_, 100 °C, 30 min, microwave; products obtained as a racemic mixture, one enantiomer represented.

Combretastatin A-4 (CA-4), a natural product stilbene, is a potent microtubule-disrupting agent that binds at the colchicine-binding site of tubulin. The design, synthesis and biochemical evaluation of a series of analogues of the microtubule-destabilising agent CA-4 is described. The monocyclic β-lactam CA-4 analogues containing halogen substituents at the C-3 position of β-lactam ring were synthesized using the Staudinger reaction (Greene, T.F.; et al. *J. Med. Chem.*, **2016**, *59*, 90–113; Malebari, A.M., et al. *Eur. J. Med. Chem.* **2017**, *130*, 261–285). Previous investigations described two approaches for the construction of 3-fluoro-β-lactams using the ketene-imine condensation or the enolate-imine condensation method (Araki, K., et al. *Tetrahedron Lett.* **1991**, *32*, 5461–5464). In the present work, the synthesis of 3-fluoro and 3,3-difluoro substituted β-lactams was developed easily by a convenient microwave assisted Reformatsky reaction using ethyl bromofluoroacetate and ethyl bromodifluoroacetate, respectively (Scheme 2). To the best to our knowledge, this is the first report of this new synthetic approach for 3-fluoro and 3,3-difluoro β-lactams as CA-4 analogues. The reaction was successful with short reaction time compared to the conventional Staudinger reaction, with moderate yield and few steps required, and it was demonstrated to be a convenient and facile method for the preparation of a variety of 3-fluoro and 3,3-difluoro β-lactams. The 3-fluoro β-lactams (1–8) and 3,3-difluoro β-lactams (9–16) in this series contained the 3,4,5-trimethoxyphenyl ring A, (required for potent activity of CA-4), together with various ring B substituents. The structure of β-lactams 6 and 14 was confirmed by X-ray crystallography. Preliminary cell viability studies demonstrated the antiproliferative effects of these novel compounds, with an IC_50_ value of 0.153 µM for 6 in MCF-7 human breast cancer cell line.

### 7.3. The Influence of Fluorination on Aliphatic Lipophilicity (P03)

LinclauBrunoSchool of Chemistry, University of Southampton, Southampton SO171BJ, UK; bruno.linclau@soton.ac.uk

Fluorination of bioactive compounds is commonly employed in the drug optimisation process, given its potential to modulate a range of properties, and its ability to prevent or slow down undesired processes such as oxidation and/or acid-catalysed degradation. In this context, aliphatic fluorination (as opposed to aromatic fluorination) is receiving increasing interest. Our group is interested in investigating how fluorination affects molecular conformation (Bogdan, E., et al. *Chem. Eur. J.* **2015**, *21*, 11462–11474) and lipophilicity ((a) Linclau, B., et al. *Angew. Chem. Int. Ed.* **2016**, *55*, 674–678 (*VIP*); (b) Effries, B., et al. *J. Med. Chem.* **2018**, *61*, 10602–10618), as well as hydrogen bond properties of adjacent functional groups including chiral aliphatic structures and carbohydrates ((a) Graton, J., et al. *Angew. Chem. Int. Ed.* **2012**, *51*, 6176–6180 (*VIP*). (b) Graton, J., et al. *Chem. Eur. J.* **2017**, *23*, 2811–2819) and how this impacts on binding affinity ((a) van Straaten, K.E., et al. *J. Am. Chem. Soc.* **2015**, *137*, 1230–1244; (b) N′Go, I., et al. S. *Chem. Eur. J.* **2014**, *20*, 106–112).

The introduction of fluorine on aliphatic (sp^3^) carbon often leads to a reduction in lipophilicity, and is the result of a number of effects including hydrophobic surface and dipole introduction, as well as reduction of polarizability. The importance of these opposing effects depends also on a number of factors such as absolute lipophilicity and conformational effects, the latter of which can, in turn, be very different in water and octanol.

We developed a convenient ^19^F NMR-based method for the log*P*-determination of fluorinated compounds, thus not relying on UV-activity, and used this methodology for an extensive study of aliphatic lipophilicity based on an alkanol scaffold.

This presentation will give an overview of these, and other, unpublished findings, and will involve an extensive list of aliphatic fluorination motifs.

### 7.4. Exploring the Impact of Polymer Size on the Biological Activity of PEG-Haloperidol Conjugates (P04)

NatfjiAz AlddienPegoraroCamillaWatsonKimOsbornHelenGrecoFrancescaSchool of Pharmacy, University of Reading, Whiteknights PO BOX 224 Reading RG6 6AD Berkshire, UK; c.pegoraro@student.reading.ac.uk

**Figure 6:**
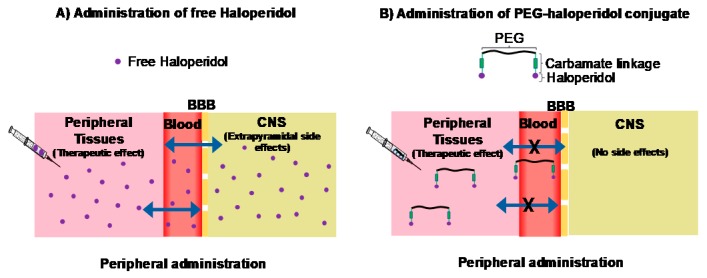
Comparison of the peripheral administration of free haloperidol (HA) and PEG-HA conjugate.

Haloperidol (HA) is a well-known antagonist of D_2_ receptors, clinically used as an antipsychotic. Moreover, HA has been shown to be a ligand of different types of receptors (centrally and peripherally) including σ receptors, which have a role in the growth and development of different types of solid tumours (van Waarde, A., et al., *Biochim. Biophys. Acta Biomembr*. **2015**, *1848*, 2703–2714). However, HA, as a small molecule, has the ability to permeate across the blood–brain barrier (BBB) and, consequently, produces central nervous system (CNS) (extrapyramidal) side effects, which limits its peripheral clinical applications. We have previously suggested that conjugating HA to a non-biodegradable polymer through a biologically stable linker could minimise or even prevent its diffusion though the BBB while retaining its biological efficacy (Figure 6). Therefore, conjugation between PEG, a non-biodegradable polymer, and HA through a carbamate linkage (biologically stable) was developed for the first time by our group (Heath, F., et al. *Polym. Ch.* **2016**, 7204–7210). In silico and in vitro studies revealed that the conjugated HA is extremely unlikely to cross the BBB and that it maintains binding activity towards D_2_ receptors, but at low level. In the present study, we evaluated the impact of polymer size on the biological activity of PEG-HA on σ receptors in vitro. In addition to this, molecular docking studies, in σ and D_2_ receptors, were performed to understand the effect of conjugation on the binding of HA to these receptors.

PEG-HA conjugates, using different sizes of PEG (6000, 2000 Da), were synthetized and characterised. A two-step synthesis was carried out: first, HA was modified to have an amine handle that allows conjugation; second, the HA-amine handle was conjugated to NHS-activated PEG. In vitro cytotoxicity studies, via σ receptors, were performed on MCF-7. The IC_50_ values of free HA and PEG-HA conjugates were determined using the MTT assay. In parallel, molecular docking studies were conducted. The results revealed that conjugating HA to PEG affected its binding to the receptors. In the case of D_2_ receptors, the position of PEG-HA inside the pocket was different compared to free HA while retaining the salt bridge interaction with Asp114, which has been indicated to be essential for the biological activity. These findings might justify the reduced biological activity observed for PEG-HA in vitro. Regarding the σ receptors, initial results indicated that conjugating HA to PEG influenced its binding to these receptors.

### 7.5. Design, Synthesis and Evaluation of Novel PqsR Inhibitors as Adjuvant Therapy to Treat Pseudomonas aeruginosa Infections (P05)

MashabiAlaa^1^SoukariehFadi^2^RichardsonWilliam^1^KellamBarrie^1^CámaraMiguel^2^StocksMichael^1^1School of Pharmacy, Nottingham NG7 2RD, UK2School of Life Sciences, National Biofilms Innovation Centre, Centre for Biomolecular Sciences, University of Nottingham, Nottingham NG7 2RD, UK; alaa.mashabi@nottingham.ac.uk, fadi.soukarieh@nottingham.ac.uk

**Figure 7:**
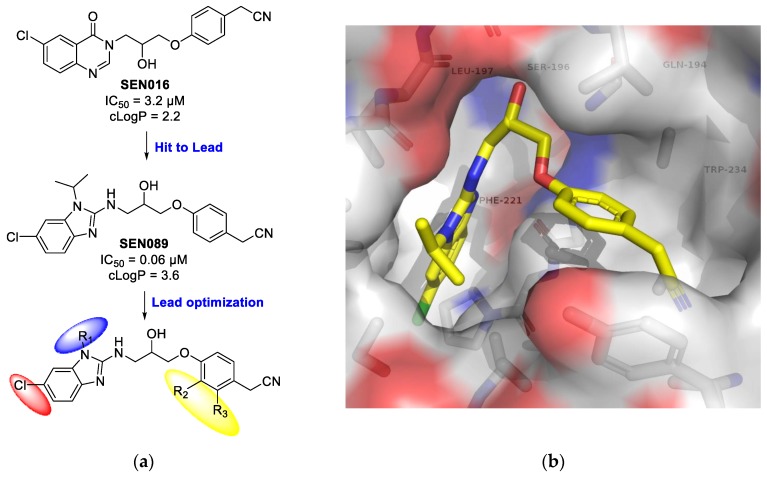
(**a**) Improvement in the activity from SEN016 (hit) to SEN089 (lead) and the structure–activity relationship (SAR) study of SEN089 based on the modifications of R_1_, R_2_ and R_3_. (**b**) The co-crystal structure of SEN089 complexed with PqsR^CBD^ domain.

**Abstract** Quorum sensing (QS) is a cell-to-cell communication mechanism used by bacterial populations to control virulence traits and resistance mechanisms (Williams, P., et al. *Philos. Trans R SocLond. B Biol. Sci.* **2007**, *362*, 1119–1134). *P. aeruginosa* has several QS systems, one of which, the *Pseudomonas* quinolone system (*pqs*) uses alkyl-quinolone (AQ)-derived QS molecules. PqsR is the key regulatory protein responsible for the activation of the *pqs* system upon interaction with the *P. aeruginosa* ligands (2-heptyl-3-hydroxy-4(1H)-quinolone (PQS) and 2-heptyl-4-hydroxyquinoline (HHQ)) to drive the upregulation of AQ production (feedback activation) and virulence factors biosynthesis (Ilangovan, A., et al. *PLoS Pathog.* **2013**, 9(7), e1003508). It has been shown that the inhibition of the PqsR receptor attenuates bacterial pathogenicity, without affecting bacterial growth and viability. Our aim was to design, synthesise and evaluate a series of new PqsR inhibitors using a quinazolinone-derived molecule (SEN016, IC_50_ 3.2 µM) that was found during a high-throughput screening (HTS) (Figure 7). Replacement of the quinazolinone ring by benzimidazole (SEN089) enhanced the activity (IC_50_ 0.067 µM). However, further lead optimisation was required to enhance its biological and physiochemical profile. The co-crystal structure of SEN089 with PqsR^LBD^ was determined and used to guide the lead optimisation strategy. Herein, we report further SAR analysis around modifications (R_1_, R_2_ and R_3_) of SEN089, which resulted in a series of novel PqsR inhibitors with enhanced biological activity and improved physiochemical properties.

### 7.6. A Novel in Vivo Anti-Amnesic Agent, Specially Designed to Express Both Acetylcholinesterase (AChE) Inhibitory, Serotonergic Subtype 4 Receptor (5-HT_4_R) Agonist and Serotonergic Subtype 6 Receptor (5-HT_6_R) Inverse Agonist Activities, with a Potential Interest against Alzheimer′s Disease (P06)

HatatBérénice^1^,^2^YahiaouiSamir^1^LecouteyCédric^1^DavisAudrey^1^FreretThomas^3^BoulouardMichel^3^ClaeysenSylvie^2^RochaisChristophe^1^DallemagnePatrick^1^1Normandie Université, UNICAEN, Centre d′Etudes et de Recherche sur le Médicament de Normandie (CERMN), Caen, France2IGF, University of Montpellier, CNRS, INSERM, Montpellier, France3Normandie Université, UNICAEN, INSERM, U1075, GIP CYCERON, COMETE, Caen, France; cedric.lecoutey@unicaen.fr

This work describes the conception and synthesis in vitro and in vivo biological evaluation of novel multi-target directed ligands (MTDL) able to both activate 5-HT_4_ receptors, block 5-HT_6_ receptors and inhibit acetylcholinesterase activity (AChE), in order to exert a synergistic anti-amnesic effect, potentially useful in the treatment of Alzheimer′s disease (AD). Indeed, both activation of 5-HT_4_ and blockage of 5-HT_6_ receptors led to an enhanced acetylcholine release, suggesting that it could lead to efficient restoration of the cholinergic neurotransmission deficit observed in AD. Furthermore, 5-HT_4_ receptor agonists are able to promote the non-amyloidogenic cleavage of the amyloid precursor protein (APP) and to favour the production of the neurotrophic protein sAPPα. Finally, we identified a pleiotropic compound, [1-(4-amino-5-chloro-2-methoxyphenyl)-3-(1-(3-methylbenzyl)piperidin-4-yl)propan-1-one fumaric acid salt, which displayed in vivo an anti-amnesic effect in a model of scopolamine-induced deficit of working memory at a dose of 0.3 mg/kg.


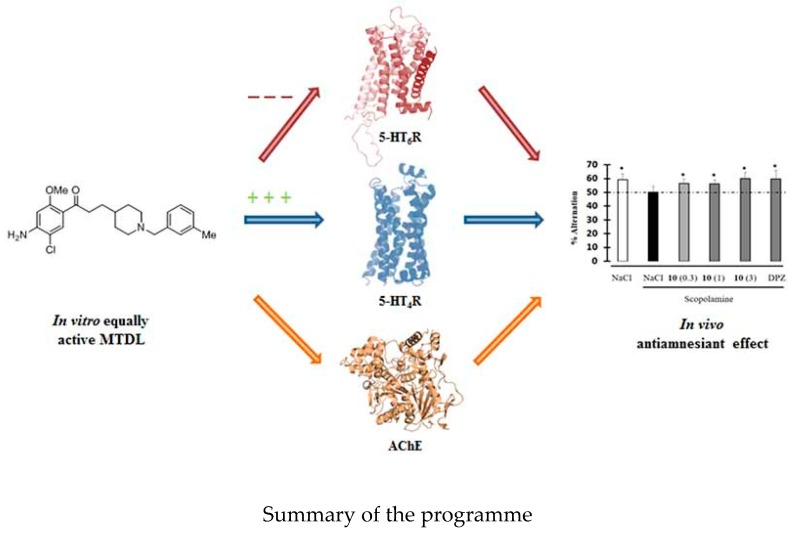


This work was supported by funding from the Fondation Vaincre Alzheimer (#FR-15072) and the Fondation Plan Alzheimer (AAP2015 Project TRIAD 016).

### 7.7. Exploring Brain–Immune Interaction with Hyper-Polarised Xenon-129 Magnetic Resonance Imaging (P07)

VigierClément^1^DubostEmmanuelle^1^VivienDenis^2^FernandezChristian^3^CaillyThomas^1^,^4^,^5^FabisFrederic^1^1Normandie Univ, UNICAEN, Centre d′Etudes et de Recherche sur le Médicament de Normandie (CERMN), 14000 Caen, France2Physiopathology and Imaging of Neurological Disorders (PhIND), 14000 Caen, France3Laboratoire Catalyse et Spectrochimie (LCS), 14000 Caen, France4Normandie Univ, UNICAEN, IMOGERE, 14000 Caen, France5Department of Nuclear Medicine, CHU Côte de Nacre, 14000 Caen, France; clement.vigier@unicaen.fr

Inflammation is a hallmark of most neurological disorders and the ability to detect, quantify and monitor the inflammatory response of the central nervous system (CNS) could have large implication for both diagnosis and therapeutic response prediction (V. Wee Yong, *Neuroscientist*. **2010**; 16: 408–20). Among the different diagnostic modalities suitable to detect neuro-inflammation, plasmatic biomarkers can be used, but no reliable and specific plasmatic biomarker of neuro-inflammation has been identified to date. Recently, molecular imaging of neuro-inflammation has been developed—the in vivo detection of a protein, the P-selectin, which is over-expressed at the luminal surface of endothelial cells during neuro-inflammation was performed using micro-sized particles of iron oxide (MPIO). However, this biosensor cannot be used in humans because of its toxicity.

Hyper-polarised xenon-129 (HP-129Xe) has recently emerged as a promising biocompatible contrast agent to improve sensitivity of MRI, successfully used to acquire images of the human pulmonary system (Liburn, D. M. L., et al. *J. Magn. Reson.* **2013**, 7, 173) and brain (Swanson, S. D., et al. *Magn. Reson. Med.* **1997**, 38, 695). However, this gas is not specific of a biological target, and therefore it has to be vectorised using a molecular host to be a valuable biosensor. Among them, cryptophanes showed very good xenon encapsulation properties, leading to a number of in vitro studies using HP-129Xe cryptophane-based biosensors reported in the literature since the 2000s (Wang, Y., et al. *Acc. Chem. Res.* **2016**, 49, 2179–2187).

**Figure 8:**
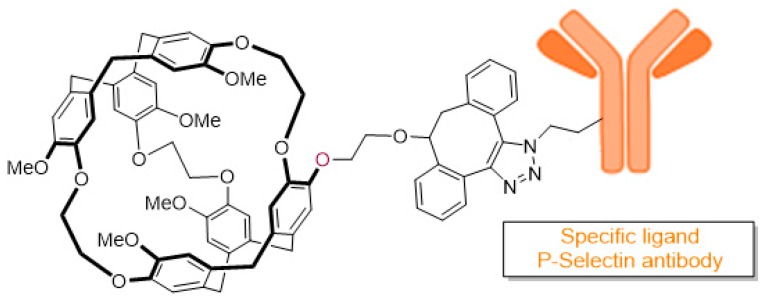
Representation of the biosensor.

Here, we propose the design and the synthesis of a new biocompatible MRI biosensor composed of a cryptophane core able to encapsulate xenon, and a well-characterized P-selectin antibody able to selectively bind endothelial P-selectin with high affinity. The two parts will be connected with a linker using copper-free click chemistry to afford the desire biosensor (Figure 8).

In this presentation, we will focus on the conception and the synthesis of the biosensor.

### 7.8. Multivalent Inhibitors of Sialidases (P08)

AssaillyCoralie^1^BrissonnetYoan^1^SaumonneauAmélie^2^TellierCharles^2^DaligaultFranck^2^GrandjeanCyrille^2^DeniaudDavid^1^GouinSébastien^1^1CEISAM, UMR CNRS 6230, UFR des Sciences et des Techniques, 2 rue de la Houssinière, BP 92208, 44322 Nantes Cedex 32UFIP, UFR des Sciences et des Techniques, 2 rue de la Houssinière, BP92208, 44322 Nantes Cedex 3; coralie.assailly@univ-nantes.fr

In recent years much effort has been devoted to the design of potent and selective glycosidase inhibitors. However, potential candidates often lack of glycosidase selectivity, and non-specific inhibitions may lead to severe side-effects. Limiting selectivity issues is a challenge unmet with the first generation of inhibitors.

Sialidases are glycosidases involved in many physiological and pathological functions. Many viruses, bacteria and parasites produce this enzyme to cleave sialic acid residues from host cells, unmasking membrane receptors for adhesion and invasion (Newstead, S. L.; et al. *J. of Biol. Chem.* **2008**, *283* (14), 9080–9088). There are several classes of sialidases, some display a unique catalytic site (CAT) to cleave the sialosides, whereas others have an additional lectinic site called CBM (carbohydrate-binding module), which is an anchoring point for glycans, thus increasing the catalytic efficiency of the enzyme (Thobhani, S., et al. *J. Am. Chem. Soc.* **2003**, *125* (24), 7154–7155). These sialidases with CAT and CBM are virulence factors common to many protozoal and pathogenic bacteria, and are potential therapeutic targets. Furthermore, human sialidases involved in important physiological functions do not express CBMs.

In this work, we developed an alternative approach to the “lock and key concept” to achieve high affinities and selectivities for sialidases (Brissonnet, Y., et al. *Chem. Eur. J.* **2019**, *25* (9), 2358–2365). Multivalent thiosialosides were designed to bind to both the CAT and the CBM of pathogenic sialidases.

**Figure 9:**
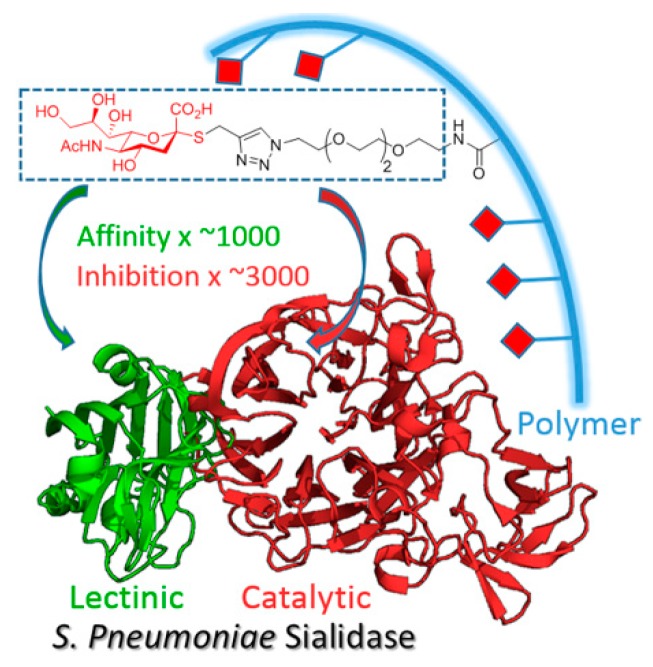
Inhibition by multivalent compound.

Sialidases from *Vibrio cholerae*, *Trypanosoma cruzi* and *Streptococcus pneumoniae* were produced with and without the CBM to determine the importance of dual targeting and the validity of the multivalent concept (Figure 9).

Inhibition tests of a polyvalent thiosialoside against the representative bacterial, parasitic and fungal sialidases showed inhibitory activity up to the nanomolar range with a strong synergistic effect. These results extend the multivalent concept to this important class of enzymes for which transition-state inhibitors failed to reach the submicromolar level.

### 7.9. Discovery of Highly Selective PI3K δ Inhibitors for Treatment of Blood Cancers and Autoimmune Diseases (P09)

BuzriedaAnas^1^MacdonaldSimon^2^MistryShailesh^1^StocksMichael J.^1^1Department of Pharmacy, University of Nottingham, Nottingham NG7 2QL, UK2GSK, Gunnels Wood Rd, SG1 2NY Stevenage, Hertfordshire; Paxab16@Nottingham.AC.Uk

**Figure 10:**
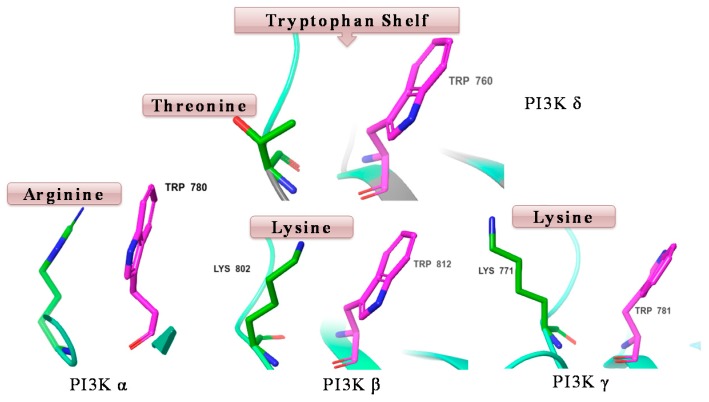
Comparison of the tryptophan region of crystal structure of PI3K δ, α, β and γ.

Phosphatidylinositol-3-kinases (PI3Ks) are lipid kinases that regulate a plethora of cellular processes including proliferation, survival and motility. PI3Ks have been divided into three classes. Of these, the most characterized is the class I enzymes, which are activated by cell surface receptors to generate phosphatidylinositol-3,4,5-trisphosphate (PIP3). Class I PI3Ks are further divided into four isoforms (α, β, γ and δ), the former two isoforms are ubiquitously expressed, whereas PI3K γ and δ are found mainly in the haematopoietic system (Schwehm, C., et al. *J. Med. Chem.* **2017**, *60*, 1534–1554). PI3K δ has been identified as an attractive drug target, where it plays a key role in the pathogenesis of blood cancer (lymphoma, leukaemia) and autoimmune disease, such as rheumatoid arthritis. Selective inhibition of PI3K δ is desired to avoid the side effects associated with the inhibition of PI3k α and β, which play a major role in insulin signalling and platelet aggregation. Idelalisib is the first approved selective PI3K δ inhibitor for the treatment of chronic lymphocytic leukaemia (CLL) and follicular lymphoma (FL). However, idelalisib has demonstrated serious side effects involving hepatic toxicity, colitis, serious diarrhoea and pneumonitis (Somoza, J., et al. *J. Biol. Chem.* **2015**, *290*, 8439–8446). Starting from a non-selective PI3K inhibitor “pictilisib”, we identified a series of potent indole-based inhibitors of PI3K δ with good selectivity versus other PI3K isoforms—the high δ selectivity of these compounds could be attributed to favourable interaction with “tryptophan shelf”. The “tryptophan shelf” defines the face of Trp760, that can be accessed only in PI3K δ due to movement of Thr750. On the other hand, in the other isoforms, the face of the tryptophan is “blocked” by basic residues that can form strong -cation interactions with Trp760 (Figure 10).

### 7.10. Development of Novel Antimicrobial Biopolymers (P10)

MullenDeclan^1^SarminAtiya^2^ConnellyJohn^2^YoungLouise^1^ParkinsonJohn A.^3^MoreiraVânia M.^1^,^4^1Strathclyde Institute of Pharmacy and Biomedical Sciences, University of Strathclyde, 161 Cathedral Street, Glasgow G4 0RE, UK; declan.mullen@strath.ac.uk2Centre for Cell Biology and Cutaneous Research, Barts and the London School of Medicine and Dentistry, Queen Mary University of London3Department of Pure & Applied Chemistry, University of Strathclyde, 295 Cathedral Street, Glasgow G1 1XL, UK4Drug Research Program, Division of Pharmaceutical Chemistry and Technology, Faculty of Pharmacy, University of Helsinki, Finland, Viikinkaari 5 E, P.O. Box 56, FI-00014 University of Helsinki

The decline in discovery of novel antimicrobial compounds over the last two decades has become a worldwide problem (Spellberg, B., et al. *Clin. Infect. Dis.*, **2004**, *38(9)*, 1279–1286). With the ability of microorganisms to evolve and adapt to survive exposure to antibiotics, that is, the emergence of antimicrobial resistance (AMR), human health is severely threatened (O′Neill, J.; **2014** *(Accessed: May 2019)*). For instance, AMR hampers the treatment of post-surgery infections caused by bacteria such as *Staphylococcus aureus*, and severely complicates the healing of infected chronic wounds.

Previous work in our group showed that amino acid-functionalised dehydroabietic acid derivatives are capable of killing planktonic bacteria and limit their ability to form biofilms (a. Manner, S., et al. *Eur. J. Med. Chem.* **2015**, *102*, 68–79; b. Helfenstein, A., et al. *Bioorg. Med. Chem*, **2017**, *25*, 132–137; c. Fallarero, A., et al. *Int. J. M ol. Sci.* **2013**, *14*, 12054–12072). This is particularly relevant, as biofilms’ contribution to AMR are highly resilient to traditional antimicrobials (a. Manner, S., et al. *Eur. J. Med. Chem.* **2015**, *102*, 68–79; b. Helfenstein, A., et al. *Bioorg. Med. Chem*, **2017**, *25*, 132–137; c. Fallarero, A., et al. *Int. J. Mol. Sci.* **2013**, *14*, 12054–12072). The group has also shown that the functionalisation of nanocellulose with the abietane dehydroabieylamine resulted in innovative and cost-efficient eco-friendly surfaces with antimicrobial properties and good biocompatibility (Hassan, G, et al. *ACS Sust. Chem. Eng.*, **2019**, *7*, 5002–5009). As the primary structural component of the plant cell wall, cellulose is the most common organic compound on the planet. It can be harvested and processed from many different renewable plant sources such as wood and cotton (a. Schultz, C., et al. *ACS Sustainable Chem. Eng.*, **2018**, *67*, 8317–8324; b. Lin, N., et al. *Molecules*, **2018**, *23(10)*, 2684–2708). This combination of renewability, low cost, strong structural integrity and its innate non-cytotoxic properties make it an ideal basis for development of new antimicrobial biomaterials.

The goal of our current work is to build from this knowledge and chemically modify medically relevant biopolymers with our in-house library of antimicrobial abietanes to produce materials with innate antibacterial properties and explore their potential applications in wound-healing.

Acknowledgements: Tenovus Scotland, The Engineering and Physical Sciences Research Council (EPSRC)

### 7.11. Stories from Staudinger: Synthesis of Chiral Beta-Lactams (P11)

O′BoyleNiamhMeeganMaryMcLoughlinEavan1School of Pharmacy and Pharmaceutical Sciences, Trinity College, University of Dublin, Dublin, Ireland; mclougea@tcd.ie

**Figure 11:**
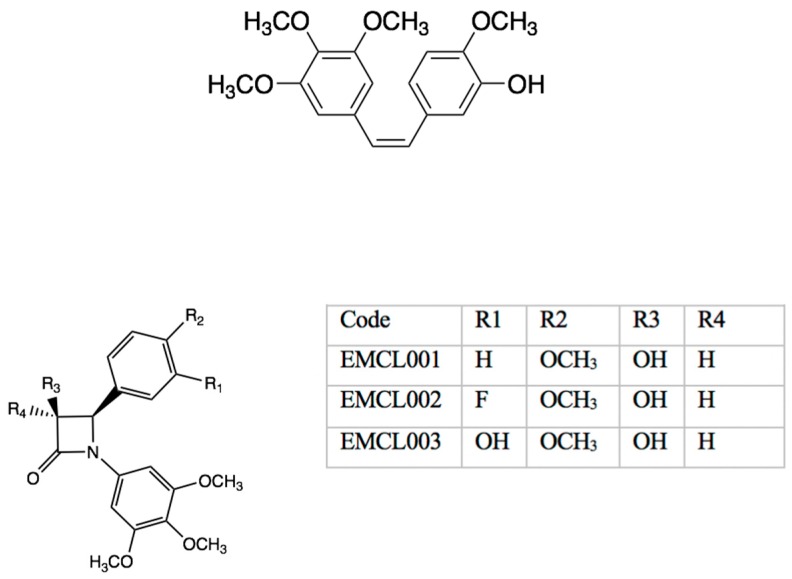
Structures of discussed compounds

Combretastatin A-4 (CA-4) is the most potent antimitotic agent of the combretastatin A series, a group of diaryl stilbenes isolated from the wood of the South African tree *Combretum caffrum* (Watt, J. M., et al. *The Medicinal and Poisonous Plants of Southern and Eastern Africa*. E. & S. Livingstone Ltd.: Edinburgh and London, United Kingdom, **1962**). It has significant anticancer activity through inhibition of tubulin polymerization and microtubule assembly (Pettit, G. R., et al. *Experientia* **1988**, 45). While *cis* stilbene structures demonstrate superior biological activity, the corresponding *trans* derivatives are inherently more stable. Isomerization of *cis* CA-4 to the *trans* form is observed both during storage and in vivo during metabolism, which dramatically reduces anti-tumour activity (Ohsumi, K., et al. *Bioorg. Med. Chem. Lett.* **1998**, *8*, 3153–3158). Our group previously employed the Staudinger reaction to synthesize novel 3-hydroxy-1,4-diaryl-2-azetidinones. The problem of CA-4′s *cis–trans* isomerization has been overcome via chemical manipulation of CA-4′s alkene bridge, utilizing a β-lactam ring to induce *cis* restriction. A number of analogues (Figure 11) have shown potent nanomolar antiproliferative activity in MCF-7 and HL-60 cells with enhanced activity relative to CA-4 (O′Boyle, N. M., et al. *J. Med. Chem*. **2010**, *53*, 8569; Azizah, M., et al. *Eur. J. Med. Chem.* **2017**, *130*, 261–285). Typical Staudinger reactions form mixtures of *cis* and *trans* isomers depending on reaction conditions employed, and additionally, at the 3-hydroxy position′s chiral center, racemic mixtures of R&S enantiomers. *Trans* isomers of 3-substituted-2-azetidinones have been shown to be up to 50 times more potent than the corresponding *cis* derivatives emphasizing the requirement to optimize the Staudinger approach to minimize yields of the undesirable *cis* isomer. Levo- and dextro-rotatory enantiomers hold potential to display lesser or greater biological activity relative to one another.

Our current work aims to (1) improve the available yield for chiral resolution by determining the necessary conditions to achieve stereoselective synthesis of *trans* 3-hydroxy-1,4-diaryl-2-azetidinones in the Staudinger reaction and (2) provide purification of racemic mixtures using *N*-(tert-butoxycarbonyl)-L-Proline as a chiral resolving agent to afford optically pure enantiomers for further biological evaluation. *Trans* 3-hydroxy β-lactams have been separated from *cis* derivatives using chromatographic purification. We have since optimized the Staudinger reaction to return relative yields of 95% relative ratio of *trans*/*cis* isomers, as indicated by integration of protons at position 3 and 4 of the β-lactam on ^1^H-NMR. Diastereomeric resolution using flash column chromatography followed by hydrolysis of chiral resolving agents successfully yielded enantiomers of EMCL001 and 2 (Figure 11). Preliminary biochemical data for the enantiomers in breast cancer cells will be reported.

### 7.12. Promising Biologically Active Peptides of Hirudo medicinalis (P12)

GrafskaiaEkaterina^1^,^2^NadezhdinKirill^2^,^3^TalyzinaIrina^3^,^4^PolinaNadezhda^1^PodgornyOleg^1^,^5^BobrovskyPavel^1^LatsisIvan^1^ManuveraValentin^1^,^2^LazarevVassili^1^,^2^1Federal Research and Clinical Center of Physical-Chemical Medicine of Federal Medical Biological Agency, 119435 Moscow, Russia2Moscow Institute of Physics and Technology (State University), 141701 Dolgoprudny, Russia3M.M. Shemyakin and Yu.A. Ovchinnikov Institute of Bioorganic Chemistry, Russian Academy of Sciences, 117997 Moscow, Russia4Federal State Budget Educational Institution of Higher Education M.V.Lomonosov Moscow State University (Lomonosov MSU or MSU), 119991 Moscow, Russia5Koltzov Institute of Developmental Biology of Russian Academy of Sciences, 119991 Moscow, Russia; grafskayacath@gmail.com

Our previous research aimed at complete genome sequence of medicinal leech *Hirudo medicinalis*, allowing for in silico implementation of the searching algorithms to identify new biologically active peptides, such as thrombolytic, anticoagulant and antibacterial agents. We applied computational algorithms to the medicinal leech *Hirudo medicinalis* genome assembly and identified homologs of serine proteinase inhibitors, promising anticoagulant proteins and candidate antimicrobial peptides (AMPs). Identified AMPs were chemically synthesised and assayed antimicrobial activity against three bacterial species (*Escherichia coli, Bacillus subtilis and Chlamydia thrachomatis*), cytotoxic and hemolytic activities and determined their secondary structures by NMR spectroscopy. Eight peptides exhibited antimicrobial activity, and two peptides, 3967 and 536–1, reduced the survival of *E. coli, B. subtilis* and *C. thrachomatis* cells through the disruption of cellular integrity at a concentration of 10 µM. Almost all the examined AMPs exhibited low-to-moderate haemolytic activity with no effects on the viability of cultured eukaryotic cells. Moreover, it was shown that three peptides, 536_2, 12530 and 3967, adopted α-helical conformation in membrane-mimetic DPC micelles. Overall, our experimental data verify the utility of the developed computational algorithms for the discovery of potential biologically active peptides. Using developed computational algorithm, we identified two promising medicinal leech AMPs, 3967 and 536–1, which are able to be considered in perspective as therapeutic agents.

*This work was supported by the Russian Science Foundation (project №17–75–20099).*

### 7.13. Subtype-Selective Fluorescent Ligands as Pharmacological Tools for the Human Adenosine hA_2A_ AR Receptor (P13)

ComeoEleonora^1^,^3^KindonNicholas D.^1^,^3^SoaveMark^2^,^3^StoddartLeigh A.^2^,^3^HillStephen J.^2^,^3^KellamBarrie^1^,^3^1Division of Biomolecular Sciences and Medicinal Chemistry, School of Pharmacy, University of Nottingham, NG7 2RD, UK2Division of Physiology, Pharmacology and Neuroscience, School of Life Sciences, University of Nottingham, NG7 2UH, UK3Centre of Membrane Proteins and Receptors (COMPARE) University of Birmingham and Nottingham, UK; eleonora.comeo@nottingham.ac.uk

The last decades have witnessed a significant growth and development of fluorescence-based techniques as a means to facilitate understanding of the signalling and dynamics of drug targets such as G protein-coupled receptors (GPCRs) in their native cellular environment (Vernall, A. J, et al. *Br. J. Pharmacol.* **2014**, *171* (5), 1073–1084; Stoddart, L. A., et al. *Sci. Rep.* **2018**, *8* (1), 1–19). Among the class A GPCRs, the human adenosine hA_2A_AR receptor represents an attractive drug target, which has been the subject of intensive medicinal chemistry research during the last 40 years due to the broad tissue distribution and implication in modulating a variety of biological processes (Ruiz, M., et al. *J. Med. Chem.* **2014**, *57*, 3623–3650). The hA_2A_AR mainly signals through the stimulatory GPCR and, upon receptor activation, adenyl cyclase is stimulated, leading to Cyclic adenosine monophosphate cAMP accumulation. The therapeutic potential of targeting the hA_2A_AR has been broadly investigated, leading to the development of an extensive number of synthetic agonists and antagonists for the treatment of several diseases including cardiovascular, inflammatory and CNS disorders. More recently, targeting the hA_2A_AR has generated a great interest in cancer immunotherapy. The tumour micro-environment features high concentrations of immunosuppressive adenosine that, upon A_2A_AR engagement, silences the immune response in the host, leading to tumour growth and proliferation. Indeed, A_2A_AR antagonism is being investigated as a therapeutic approach to defeat the immune system evasion by the tumour (Leone, R. D., et al. *J. Immunother. Cancer* **2018**, *6* (1), 1–9). In this regard, understanding the complexity of the adenosinergic signalling is an essential prerequisite to address and develop more efficacious therapies. This can be facilitated by exploiting fluorescent probes selectively targeting the hA_2A_AR both in vitro and in vivo (Vernall, A. J., et al. *iScience* **2018**, *6*, 280–288).

Herein, we report the design synthesis and pharmacological evaluation of two generations of subtype-selective fluorescently-labelled hA_2A_AR antagonists based on preladenant. Of particular interest for in vivo studies was the second generation of ligands, which features water soluble probes and, therefore, might display better stability in plasma and improved detection after IV-administration. Molecular modelling analysis has corroborated previously reported SAR studies indicating the optimal position for fluorophore attachment. The newly synthesised fluorescent probes retained functional activity and binding affinity at the hA_2A_AR, displaying a selectivity of >1000 nM over the other receptor subtypes. The effectiveness of the probes as pharmacological tools was also investigated in high resolution confocal imaging studies, revealing specific co-localisation with SNAP-hA_2A_AR at the cell membrane, which was surmounted by incubating the cells with high concentration of unlabelled ligand. In conclusion, given their excellent pharmacological and photochemical properties, the reported novel fluorescent ligands embody valuable tools to study the signalling and dynamics of the hA_2A_AR, not only in vitro but also in vivo, providing a robust and specific platform for further drug discovery investigations.

### 7.14. Synthesis and Pharmacological Evaluation of 5,6,7,8-Tetrahydroimidazo[1–a]Pyrazine-Based Thiosemicarbazones against Trypanosoma cruzi (P14)

de Araújo NetaMarlene S.^1^,^2^CoutinhoFelipe N.^1^,^2^de FariaAntônio R.^2^PicotCarine^1^PagniezFabrice^1^PapePatrice Le^1^SilvaTeresinha G. da^3^MarchandPascal^1^1Université de Nantes, Cibles et médicaments des infections et du cancer, IICiMed, EA 1155, F-44000 Nantes, France2Department of Pharmaceutical Sciences, University of Pernambuco, Recife-PE 50740–520, Brazil3Department of Antibiotics, University Federal of Pernambuco, Recife-PE 50740–520, Brazil; pascal.marchand@univ-nantes.fr

**Figure 12:**
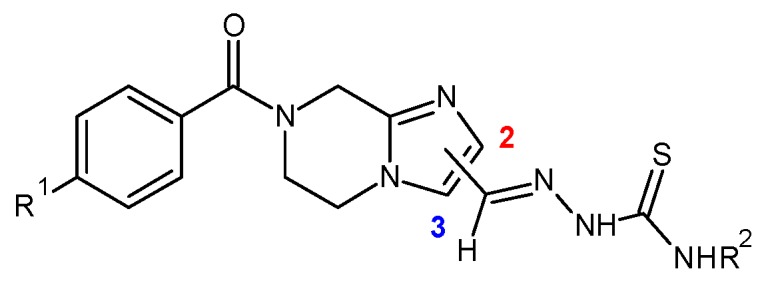
General structure of target compounds.

Neglected tropical diseases (NTDs) affect mainly underdeveloped or developing countries located in Africa, Asia and Latin America. Worldwide, there are more than one billion people affected by these diseases (World Health Organization, Neglected Tropical Diseases. Access: 26 May 2017. Information site: http://www.who.int/neglected_diseases/diseases/en/). These include Chagas disease, also known as American trypanosomiasis, a potentially life-threatening illness caused by the protozoan parasite *Trypanosoma cruzi* (*T. cruzi*). Difficulty of treatment due to the amount of side effects of the drugs currently used, the resistance of the parasite and late detection are concerns to address. Therefore, the discovery of new substances for the treatment of Chagas disease is extremely necessary. Thus, we developed a medicinal chemistry program dealing with the synthesis of 5,6,7,8-tetrahydroimidazo [1, 2-*a*] pyrazine-based thiosemicarbazones as antiparasitic agents (Moreno-Rodríguez, A., et al. *Eur. J. Med. Chem.* **2014**, *87*, 23–29; Leite, F., et al. *Eur.J. Med. Chem.* **2016**, *123*, 639–648; Marchand, P., et al. *Eur.J. Med. Chem*. **2015**, *103*, 381–395.) (Figure 12).

The first developments and biological results of this novel series of molecules will be discussed.

The authors would like to acknowledge Capes/Cofecub Program France-Brazil Me 865–15 for financial support.

### 7.15. Synthesis and Biological Evaluation of Inhibitors Targeting Signal Transducers and Activators of Transcription 5 (STAT5) Proteins in Myeloid Leukemias (P15)

PolomskiMarion^1^Brachet-BotineauMarie^2^JuenLudovic^1^GouilleuxFabrice^2^Viaud-MassuardMarie-Claude^1^PriéGildas^1^1Team IMT, GICC EA 7501, University of Tours, Labex SYNORG, 37200 Tours, France2Team LNOx, GICC ERL 7001 CNRS, University of Tours, 37032 Tours, France; gildas.prie@univ-tours.fr

Myeloid leukemias are myeloproliferative diseases that affect hematopoietic stem cells (HSC), and are divided in two types acute (AML) and chronic (CML) according to a fast or slower cell growth.

CML is mainly due to the t(9,22) genomic translocation-derived BCR-ABL fusion oncogene coding for the tyrosine kinase Bcr-Abl, which activates the transcription factors STAT5 (signal transducers and activators of transcription 5). The latter plays a crucial role in the initiation and maintenance of CML and mediates resistance to Bcr-Abl kinase inhibitors such as imatinib mesylate (IM, Glivec) (Hoelbl et al. *EMBO Mol. Med.* **2010**, *2*, 98–110; Kavalerchik, et al. *J. Clin. Oncol.* **2008**, *26*, 2911–2915). AML results mainly from internal tandem duplication (Itd) mutations in the juxtamembrane region or point mutation in fms like tyrosine kinase 3, FLT3. This oncoprotein FLT3-Itd (internal tandem duplication) has a tyrosine kinase activity, which activates STAT5 (Birkenkamp, et al. *Leukemia* **2001**, *15*, 1923–1931).

As a result, inhibiting STAT5 would contribute to reduce the survival of CML and AML cells and moreover tackle their potential chemoresistance.

**Figure 13:**
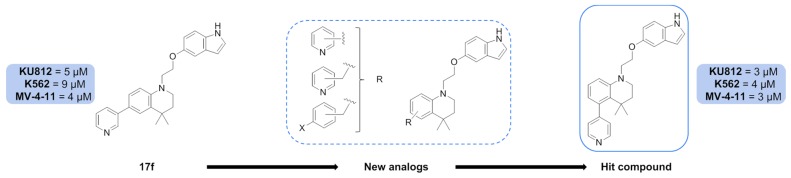
Lead **17f**, new analogs and hit compound.

A first structure–activity relationship study allowed us to identify one compound, **17f** (Figure 13), which inhibited the growth of AML and CML cell lines as well as phosphorylation and transcriptional activity of STAT5. These results suggest that **17f** might be a new lead molecule targeting STAT5 signalling in myeloid leukemias (Juen, et al. *J. Med. Chem.* **2017**, *60*, 6119–6136). Thanks to these results, synthesis of **17f** new analogues with modulation around the tetrahydroquinoleine (THQ) ring were undertaken and their biological evaluation was carried out on model CML (KU812, K562) and AML (MV-4–11) cell lines. Among these new results, one compound, with the 4-pyridinyl in position 5 on the THQ ring, showed slightly better results than **17f** on all cell lines. This outcome will guide further modulation work on the THQ ring with others nitrogen heterocycles (pyridazine, triazine, etc.).

### 7.16. From Phloridzin towards Semisynthetic Bioactive Dihydrochalcones (P16)

PeraltaA. Cala^1^MayrF.^2^ViaultG.^1^TemmlV.^2^SchusterD.^3^StuppnerH.^2^SéraphinD.^1^RichommeP.^1^HélesbeuxJ-J.^1^1SONAS, EA921, UNIV Angers, SFR QUASAV, Faculty of Health Sciences, Department of Pharmacy, 16 bd Daviers, 49045 Angers Cedex 01, France2Institute of Pharmacy/Pharmacognosy and Center for Molecular Biosciences Innsbruck (CMBI), University of Innsbruck, 6020 Innsbruck, Austria3Department of Pharmaceutical and Medicinal Chemistry, Institute of Pharmacy, Paracelsus Medical University Salzburg, 5020 Salzburg, Austria; jj.helesbeux@univ-angers.fr

**Figure 14:**
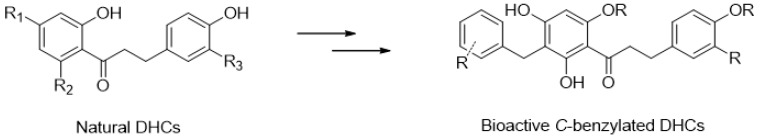
Structures of natural dihydrochalcones (DHCs) isolated from apple tree leaves, and example of semisynthetic modifications (Table 1).

Plant secondary metabolism produces a large number of specialized compounds (over 300,000) including terpenoids, alkaloids and (poly)phenols. Often referred to as “natural products” (NPs), many of these compounds are used for medicinal, cosmetical and/or nutraceutical purposes. Apple trees (*Malus x domestica* Brokh.) are widely cultivated all around the world, and apple is the first fruit to be produced in Europe. In France, the “Région des Pays de la Loire” appears as the second national producer of apples, with apple trees being the main cultivated surface in this area. Apple fruits/trees are characterized with a cell specific accumulation—in different tissues—of varying levels of bioactive polyphenols including proanthocyanidins, anthocyanins, flavonols and dihydrochalcones (DHCs) (Figure 14).

To date, around 250 DHCs are known, either as glycosylated derivatives or *C*-alkylated compounds (C. Rivière *in Studies in Natural Products Chemistry*, **2016**, *51*, 253–281). Amongst the latter, some *C*-benzylated compounds have demonstrated significant pharmacological effects (anti-inflammatory, anti-tumoral) (Somsrisa, J., et al. *Molecules* **2013**, *18*, 6898–6907; Prawat, U., et al. *Planta Med.* **2013**, 79, 83–86). Therefore this series of compounds is of high interest because of their pharmacological potential. The main goal of this project is to (semi-)synthesize original DHCs, in particular, *C*-benzylated derivatives, starting from phloridzin or sieboldin extracted from locally produced apple tree leaves as renewable sources. The semi-synthetic analogues will then be submitted to biological assays to evaluate their anti-tumoural potential.

### 7.17. Synthesis and Pharmacological Study of Alprenolol Analogues at the Secondary Conformation of the β_1_-Adrenoceptor (P17)

SousaEmanuel P.^1^,^2^ScammellsPeter J.^3^BakerJillian G.^2^MistryShailesh N.^1^1School of Pharmacy, Centre for Biomolecular Sciences, University of Nottingham, Nottingham NG7 2RD, UK2Cell Signalling, School of Life Sciences, Queen′s Medical Centre, University of Nottingham, Nottingham NG7 2UH, UK3Medicinal Chemistry, Monash Institute of Pharmaceutical Sciences, Monash University, 3052 Victoria, Australia; emanuel.pintodesousa@nottingham.ac.uk

The β_1_-adrenergic receptor (β_1_-AR) exists in at least two distinct agonist conformations: (1) a primary conformation where responses are readily inhibited by antagonists and (2) a secondary conformation of which the precise nature is unknown, where agonist responses are relatively resistant to antagonism (Kaumann, A.J., et al. *Pharmacol. Ther.* **2008**, *118*, 303–336).

Endogenous catecholamines (e.g., adrenaline) and conventional agonists (e.g., isoprenaline and cimaterol) mediate a response mainly through the primary conformation. Other ligands (e.g., CGP12177 **1**) mediate a response through the secondary conformation, even though the concentration required to stimulate this response is considerably greater than the concentration required to bind the primary conformation (Pak, M.D., et al. *J. Recept Signal Transduct Res*., **1996**, *16*, 1–23). Alprenolol (**2**) stimulates a response through both conformations and it also requires higher concentrations to activate the secondary conformation (Baker, J.G., et al. *Mol. Pharmacol*, **2003**, *63*, 1312–1321). To date, no ligand has been reported that binds the secondary conformation with higher affinity than the primary conformation. 

Alprenolol *bis* analogue **3** has been previously identified as the first sub-micromolar compound to have similar affinity for both conformations (Sousa, E.P, et al. **2018**, Poster presented at EFMC-ISMC 2018). In order to identify the molecular descriptors responsible for the interaction and activation of the secondary conformation of the β_1_-AR, several analogues were synthesised and pharmacologically characterised through competitive radioligand binding assays and cAMP reporter gene (CRE-SPAP) functional assays. In this communication, we report the affinity of these analogues for both conformations of the β_1_-AR.


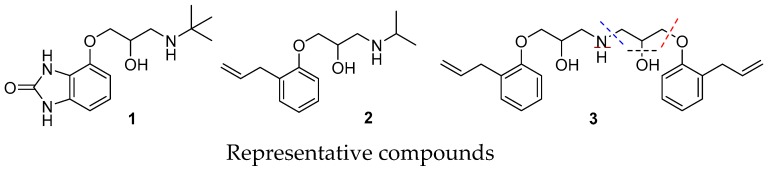


### 7.18. X-ray Diffraction Investigation of Aqueous Microgels for Drug Delivery (P18)

DolgopolovAndrey V.^1^GrafskaiaKseniia N.^2^,^3^AnokhinDenis V.^2^,^3^IvanovDimitri A.^2^,^3^,^4^PichAndrij^1^ZhuXiaomin^1^MöllerMartin^1^1DWI—Leibniz-Institute for Interactive Materials e.V. and Institute for Technical and Macromolecular Chemistry of RWTH Aachen University, D-52056 Aachen, Germany2Moscow Institute of Physics and Technology (State University), 141700 Moscow, Russia3Institute of Problems of Chemical Physics of RAS, 142432 Chernogolovka, Russia4Institut de Science de Matériaux de Mulhouse (IS2M-CNRS), F-68057 Mulhouse, France; kseniya.grafskaya@phystech.edu

**Figure 15:**
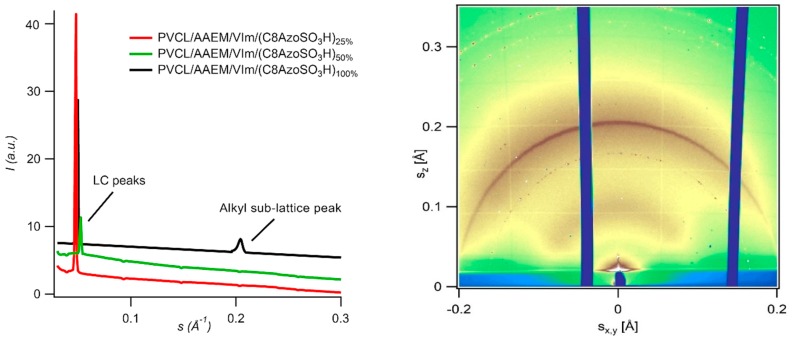
GIWAXS curves of PVCL/AAEM/VIm/(C8AzoSO_3_H) microgels with DN = 25 (red), 50 (green) and 100% (black) (**left**); GIWAXS pattern of PVCL/AAEM/VIm/(C8AzoSO_3_H) microgels with DN = 25 (**right**).

Aqueous microgels based on poly(N-vinylcaprolactam) with reversable temperature-induced volume transition are promising “smart” materials for various applications, in particular for drug delivery (Saunders, B. R., et al. *Adv. Colloid. Interfac.* **2009**, 147–48, 251–262). Here, the aqueous microgels are modified via acid–base interaction by ligand molecules. They are wedge-shaped amphiphilic sulfonic acid molecules with an azobenzene grou and alkyl chains of different lengths. Modified microgels remain colloidally stabile in water and show different responses to the change of temperature and pH within the physiologic condition (Grafskaia, K. N., et al. *Chem. Commun.* **2017**, 53, 13217). The hydrophobicity of the microgel interior increases with the increase of the modification degree. Hardening of hydrophobic nanodomains and a decrease of the hydrodynamic diameter for sample complexed with C8AzoSO_3_H at the highest neutralization degrees can be explained by formation of the alkyl sub-lattice (Figure 15).

The characterisation of local structure of microgels was performed by GIWAXS technique. In wide-angle region, a narrow reflection corresponding to d-spacing of 4.82 Å can be clearly seen. One can assume that this peak originated from the linear alkyl chain packing of the wedge-shaped mesogens (Zhu, X. M., et al. *Phys. Chem. Chem. Phys*. **2010**, 12, 1444–1452; Grafskaia, K. N., et al. *Chem. Commun.* **2017**, 53, 13217). Formation of alkyl sub-lattice can explain hardening of hydrophobic nanodomains and decrease of hydrodynamic diameter for the sample PVCL/AAEM/VIm/(C8AzoSO_3_H)_100%_.

### 7.19. DYRK2 Inhibition—A Potential Targeted Therapy for TNBC (P19)

TomkinsonNicholas C. O.^1^MackaySimon^2^BainLaura M.^1^VegaLaureano de la^3^EdwardsJoanne^4^1Department of Pure and Applied Chemistry, University of Strathclyde, Glasgow G1 1XL, Scotland2Strathclyde Institute of Pharmacy and Biomedical Sciences, University of Strathclyde, Glasgow G1 1XL, Scotland3School of Medicine, University of Dundee, DD1 9SY Dundee, Scotland4Institute of Cancer Sciences, University of Glasgow, G61 1QH Glasgow, Scotland; laura.bain@strath.ac.uk

**Figure 16:**
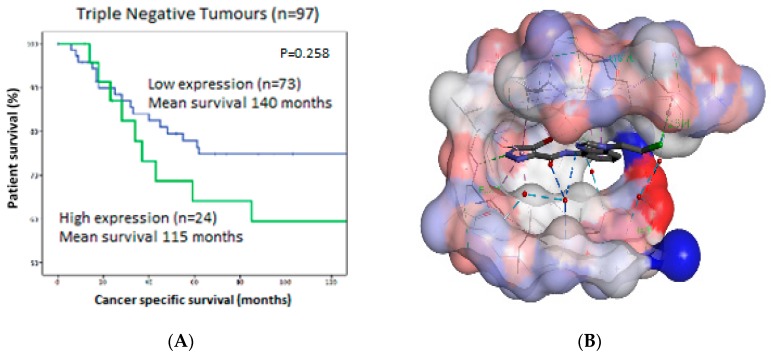
(**A**) Levels of dual specificity tyrosine(Y) regulated kinase-2 (DYRK2) expression correlate to triple negative breast cancer (TNBC) survival rate. (**B**) Model of LB35 in DYRK2 active site.

Triple negative breast cancer (TNBC) is the most aggressive form of breast cancer. It accounts for approximately 20% of all breast cancers and has an increased rate of relapse compared to non-TNBCs. This aggressive nature is due to its lack of receptors for targeted therapies and thus results in chemotherapy being the only option for treatment (Gonçalves Jr, H., et al. *Clin. Med. In. Onc.* **2018**, *12*, 1–10). This reveals the need to investigate other means for targeting and treating TNBC, more specifically, through the use of small molecule inhibitors. We discovered that dual specificity tyrosine(Y) regulated kinase-2 (DYRK2) stabilises heat shock factor-1 (HSF1), the master regulator of proteotoxic stress pathways (Rashmi, K. C., et al. *Cell Stress and Chaperones*, **2017**, *22*, 751–766). In addition, we showed that low DYRK2 expression correlates with an increased survival rate in TNBC patients (Figure 16A).

To date, there are no selective small molecule inhibitors for DYRK2. With the aid of molecular modelling, we developed an SAR profile of DYRK2 inhibitors, with our most potent inhibitor being LB35 Ki 19 nM (Figure 16B). These inhibitors revealed an impressive selectivity profile, whereby a structurally similar compound showed <30% inhibition against a panel of 40 structurally related kinases. Within this poster, we aim to describe our ongoing efforts to provide an inhibitor for DYRK2 with increased potency and examine the DMPK challenges associated with this inhibitor series.

### 7.20. Synthesis of Conformationally Restricted Adrenaline Analogs Selectively Activating β_2_-AR with Substantial Subtype Selectivity (P20)

MaulL.^1^HübnerH.^1^LiuX.^2^ShonbergJ.^1^StößelA.^1^StanekM.^1^WeikertD.^1^KobilkaB.K.^2^,^3^GmeinerP.^1^1Department of Chemistry and Pharmacy, Friedrich-Alexander University, Erlangen, Germany2Beijing Advanced Innovation Center for Structural Biology, Tsinghua University, Beijing, China3Department of Molecular and Cellular Physiology, Stanford University, California, USA; luis.maul@fau.de

The loss of a ligand′s entropy upon receptor binding can be reduced by rigidification of its chemical structure, often leading to enhanced potency, depending on whether the bioactive conformation is matched or not. Conformational restriction can also induce selectivity for receptor subtypes, as slightly different shapes of orthosteric binding pockets may require a different conformational adaption of the ligand. Taking advantage of those effects can lead to the development of novel molecular probes for scientific research and medications with improved pharmacodynamic or pharmacokinetic properties (Ring, et al. *Nature.* **2013**, *502*, 575–579).

**Figure 17:**
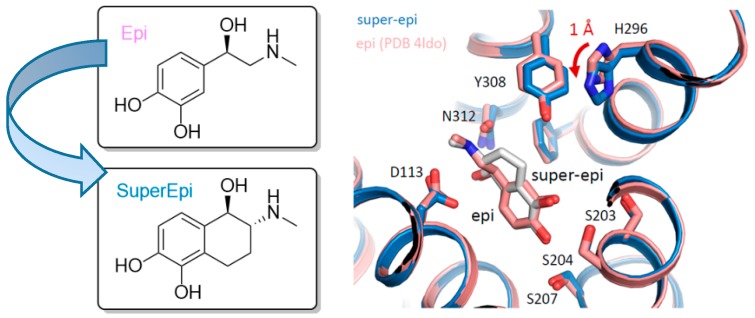
Illustration of the ß_2_-AR active state crystal structures in complex with either epinephrine or super-epi, which were shown to occupy virtually the same binding pocket in the receptor.

Using the pharmacologically important target β_2_-AR, we aimed at synthetically constraining highly flexible agonists of the catecholamine type into their bioactive conformation. Thus, we synthesized a set of eight different isomers of ethylene bridged isoprenaline, with one of them (“super-iso”) being clearly superior regarding affinity, potency and selectivity for β_2_ over β_1_. Super-epi, super-norepi, etc., refer to compounds with the same constitution and stereochemical configuration (Figure 17).

Structural insights into the receptor state and the binding poses of super-epi and super-iso were obtained by co-crystal structures of β_2_-AR bound to Nb6B9.

The poster will also give information about efficient chemical synthesis, biological investigations and structure–activity relationships (SAR) of the conformationally restricted catecholamines.

### 7.21. Synthesis of Lung Tissue Retentive Prodrugs (P21)

AyreJ.^1^SpeedD.^1^WeaverR.^2^VitulliG.^3^MorrellJ.^3^BosquillonC.^1^RedmondJ. M.^1^StocksM. J.^1^1School of Pharmacy, University of Nottingham, Nottingham, NG7 2RD2XenoGesis, BioCity, Pennyfoot Street, Nottingham NG1 1GF3DMPK, GSK, Gunnels Wood Rd, Stevenage, Hertfordshire SG1 2NY4Chemical Biology, GSK, Gunnels Wood Rd, Stevenage, Hertfordshire SG1 2NY; jack.ayre@nottingham.ac.uk, michael.stocks@nottingham.ac.uk*, joanna.m.redmond@gsk.com

The pulmonary drug delivery route possesses significant benefits over other administrative routes, including targeted delivery to the site of action, by-pass of first pass metabolism in the liver and fewer side effects owing to a reduced drug dosage. However, rapid drug elimination from lung tissue in to the hepatic vein and the pan antagonistic nature of receptor antagonists often leads to increased side effects. By exploiting the highly lung retentive nature of dibasic compounds, it is possible to reduce the rate of elimination from the lungs and thus reduce side effects. This work describes the design and synthesis of a range of dibasic prodrugs that have the potential to be lysosomally trapped within lung tissue and cleave slowly at a desired pH to effectively deliver an active drug at a slower more consistent rate.

### 7.22. Design and Synthesis of Novel Flavone Derivatives as Anti-Cancer Agents (P22)

KhaterMaiGrecoaFrancescaOsbornaHelen M.I.School of Pharmacy, University of Reading, Reading RG6 6AD, UK; m.a.a.khater@pgr.reading.ac.uk

**Scheme 3:**
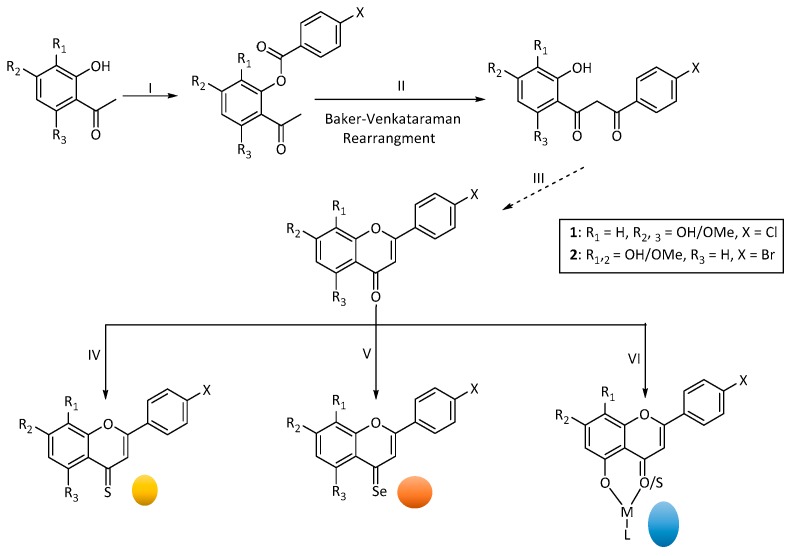
Synthesis of flavone derivatives. (**I**) Benzoyl chloride halide, 1,8-DiazaBicyclo[5.4.0]Undec-7-ene DBU, pyridine, 75 °C, 1 h; (**II**) pyridine, KOH, 50–5 °C, 2 h; (**III**) glacial acetic acid, 1% H_2_SO_4_, 90–110 °C, 1 h; (**IV**) dry toluene, Lawesson′s reagent, 110 °C, 4 h; (**V**) acetonitrile, Woollin′s reagent, microwave, 175 W, 150 °C, 5 min; (VI) MeOH, NaOMe, MLn.

Breast cancer is the most common cancer in women, resulting in 2.09 million cases worldwide (WHO Fact Sheets https://www.who.int/en/news-room/fact-sheets/detail/cancer (12 Sepetmber 2018). Flavones are one of the most active classes of flavonoids exhibiting anti-tumour activities. Flavopiridol, for example, has reached phase-II clinical trials as an anti-cancer agent against leukaemia, lymphoma and solid tumours. Selenium and ruthenium atoms have demonstrated promising anti-proliferative activities when incorporated into small molecular ligands (Olla S., et al. *Bioorg. Med. Chem. Lett.* **2009**, *19*, 1512–1516). Replacement of flavonoids′ oxo-carbonyl with a seleno-carbonyl can result in up to a 25-fold decrease in IC_50_ values on breast cancer cell lines (Martins I. L., et al. *J. Med. Chem.* **2015**, *58*, 4250–4256) (Scheme 3). On the other hand, coordination with metal atoms such as ruthenium is reported to enhance flavonoids′ water solubility up to 10-fold, which can lead to better clinical applications.

We aim to develop a new series of flavones for application against breast cancer. Two previously identified flavone leads (1 and 2 in Scheme 3 from which new selenium and ruthenium derivatives can be synthesized). The synthesis provides molecular frameworks, following a Baker–Venkataraman rearrangement pathway.. This will be followed by in vitro cytotoxicity and anti-angiogenic assessments in addition to probing the possible mechanisms of action of the most active compounds.

### 7.23. Imidazo [1, 2-a] Pyrazines Displaying In Vivo Antileishmanial Activity (P23)

BazinMarc-Antoine^1^CojeanSandrine^2^PagniezFabrice^1^NourrissonMarie-Renée^1^PicotCarine^1^CavéChristian^2^BernadatGuillaume^2^BachStéphane^3^RachidiNajma^4^LeclercqOlivier^4^LoiseauPhilippe^2^PapePatrice Le^1^MarchandPascal^1^1Université de Nantes, Cibles et médicaments des infections et du cancer, IICiMed, EA 1155, F-44000 Nantes, France2Université Paris-Sud, Université Paris-Saclay, Chimiothérapie Antiparasitaire, BIOmolécules: Conception, Isolement et Synthèse—BioCIS UMR 8076 CNRS, F-92296 Châtenay-Malabry, France3Sorbonne Universités, UPMC Paris 06, CNRS USR3151 “Protein Phosphorylation and Human Disease” group, Station Biologique, F-29680 Roscoff, France4Institut Pasteur and INSERM U1201, Unité de Parasitologie Moléculaire et Signalisation, F-75015 Paris, France; pascal.marchand@univ-nantes.fr

According to a recent report from the World Health Organization (WHO), leishmaniases— visceral leishmaniasis (VL), cutaneous leishmaniasis (CL) and mucocutaneous leishmaniasis (MCL) —collectively affect 12 million people in 98 countries, 350 million more are at risk of infection and 40,000 deaths are attributed to leishmaniases each year^.^ Currently, there are no effective vaccines and a number of drugs are used in the treatment of these parasitic infections: pentavalent antimonials, amphotericin B, miltefosine, pentamidine, paromomycin and sitamaquine (Sangshetti, J.N., et al. *RSC Adv.* **2015**, *5*, 32376–32415). Unfortunately, most of them cause side effects and high toxicities, and an inevitable resistance has developed in recent times in *Leishmania* parasites. Consequently, there is an urgent need to speed up the development of a new generation of more effective and safe antileishmanials.

In the course of our ongoing synthetic and screening programs for the obtention of new biologically active imidazo [1, 2-*a*] azines, we decided to develop bioisostere analogues of the previously described 2,3-diarylimidazo[1,2-*a*]pyridines as antileishmanial agents (Marhadour, S., et al. *Eur. J. Med. Chem.* **2012**, *58*, 543–556).

**Figure 18:**
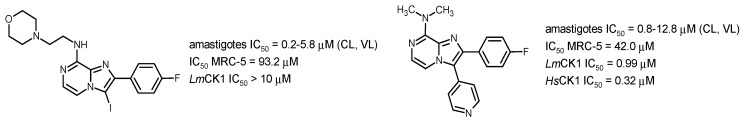
Structures of hit compounds.

Two promising analogues were highlighted as hit compounds (Figure 18), exhibiting very good in vitro and in vivo activity associated with high therapeutic index, especially on amastigote stage of the parasite (Marchand, P., et al. *Eur. J. Med. Chem.* **2015**, *103*, 381–395).

The mechanism of action involved in the antiparasitic properties will be discussed, as 4-pyridyl moiety at the C-3 position of the heterocyclic core was associated with *L. major* casein kinase 1 (*Lm*CK1) inhibition, validated as a potential molecular target for antileishmanial drug development (Durieu, E., et al. *Antimicrob. Agents Chemother.* **2016**, *60*, 2822–2833).

### 7.24. Inhibiting X-Linked Inhibitor of Apoptosis Protein (XIAP), Design of New Non-Peptidic Foldamers of SMAC (P24)

Yahia-OuahmedMezianeGiretMartinAntrayguesKévinJouanneMarieKiefferCharlineSantosJana Sopkova-de OliveiraNormandie Univ, UNICAEN, EA 4258 CERMN—FR CNRS INC3M, Caen, France

The X-linked inhibitor of apoptosis protein (XIAP) binds to and inhibits caspases 3, 7 and 9, which are some of the enzymes responsible for cell death, and therefore stopping apoptosis. XIAP is naturally regulated (inhibited) by the mitochondrial protein SMAC/DIABLO. However, XIAP is overexpressed in tumorous cells (particularly in ovarian cancer) making them resist cell death. This is why XIAP makes a suitable drug target.

The project aims at designing new non-peptidic inhibitors of XIAP in order to promote apoptotic cell death in ovarian tumours. This could be done by “mimicking” the AVPI tetra-peptide of SMAC (β strand) that is used by the latter to bind to the BIR3 domain of XIAP.

First, a pharmacophore model was built on the basis of two ligands of XIAP-BIR3 found in the literature. Then, a series of compounds were designed. The binding of these compounds to the active site was studied by molecular docking. Molecular dynamics simulations were then performed to assess the stability of the ligands in the active site. The best compounds were synthetized and tested in-vitro by fluorescence polarisation assay (FPA). The most promising candidates were selected in order to carry out new pharmacomodulations.

### 7.25. Discovery of Novel pqs Inhibitors for Pseudomonas aeruginosa Infections (P25)

LiuRui-Ling^1^SoukariehFadi^2^RichardsonWilliam^1^MistryShailesh N.^1^CámaraMiguel^2^StocksMichael J.^1^1School of Pharmacy, Nottinghamshire NG7 2RD, UK2School of Life Sciences, National Biofilms Innovation Centre, Centre for Biomolecular Sciences, University of Nottingham, Nottingham, Nottinghamshire NG7 2RD, UK; paxrll@nottingham.ac.uk

Current antibiotic treatments for *P. aeruginosa* infections have proven ineffective due to the increasing rate of resistance to them. Inhibitors of the *pqs* quorum sensing signalling, key for the control of virulence, have been regarded as a promising alternative (Melissa, S., et al. *PLoS. Pathog*. **2014**, *10*, e1004321).

**Figure 19:**
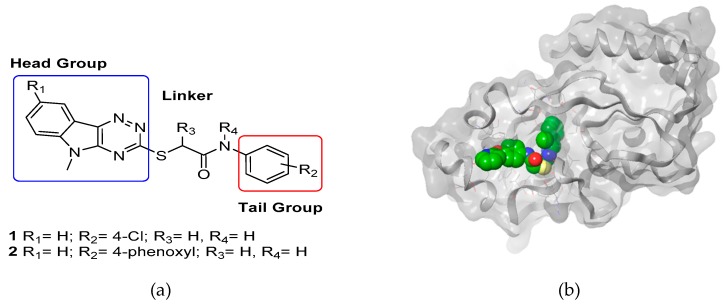
General structure of compounds and PqsR.

In this work, we report the discovery, design, synthesis and biological evaluation of a series of novel inhibitors of the PqsR regulatory protein, key for the activation of the *pqs* system, resulting in the identification of compound 2. The initial hit compound 1 (IC_50_ = 0.98 μM) was obtained through a virtual screening performed on PqsR ligand-binding domain using an in-house compound library (Figure 19a). Exploration of compound 1 gave the first generation of compounds evaluated using both PAO1-L mCTX: P*_pqsA_*-*lux* and PA14 mCTX: P*_pqsA_*-*lux P. aeruginosa pqs* bioreporter strains. Further structure activity exploration, to improve potency and physiochemical properties, gave a second generation of compounds with improved inhibitory activity. Notably, the introduction of an electron deficient tail group gave compound 2 (IC_50_ = 0.25 μM), one of the most potent known *pqs* inhibitors to date. The crystallization of compound 2 in PqsR ligand binding domain was obtained (Figure 19b) and detailed SAR studies of this series of compounds will be discussed in the poster.

### 7.26. In Silico Screening and Development of Small Molecule Neurotrophin Receptor Ligands (P26)

AntonijevicMirjanaBureauRonanDallemagnePatrickRochaisChristopheCentre d′Etudes et de Recherche sur le Médicament de Normandie (CERMN), University of Caen (UNICAEN), 14000 Caen, France; mirjana.antonijevic@unicaen.fr

Neurodegenerative diseases (ND), such as Alzheimer′s disease, Parkinson′s disease, multiple sclerosis and motor neuron disease, are on the rise worldwide. Currently, there is no cure for any ND and most of the available drugs fail to tackle ND pathogenesis. Numerous studies have been published about the role of the neurotrophin receptors in the pathogenesis of several neurodegenerative conditions (Simmons, D.A., et al. *J. Neurosci.* **2013**, *33(48)*, 18712–27). There are two types of neurotrophin receptors: a non-enzymatic, trans-membrane protein of the tumour necrosis receptor (TNFR) family—p75, and the tyrosine kinase receptors (Trks) A, B and C. Trk receptors are activated, specifically, and with high affinity, by nerve growth factor (NGF) (TrkA), brainderived growth factor and neurotrophin 4/5 (BDNF and NT-4/5) (TrkB), and neurotrophin 3 (NT3) (TrkC), whereas p75 is activated by all mentioned neurotrophins (Meldolesi, J., et al. *Pharmacol. Res.* **2017**, *121*, 129–137). The implication of the TrkB receptor in the pathogenesis of several neurodegenerative conditions (Alzheimer′s, Parkinson′s, and Huntington′s disease) point to the therapeutic potential of TrkB agonists (Nomura, T., et al. *J. Neurosci.* **2017**, *37(47)*, 11298–11310; Zheng, H., et al. J. *Metab. Brain Dis.* **2018**, *33*, 1961).

**Figure 20:**
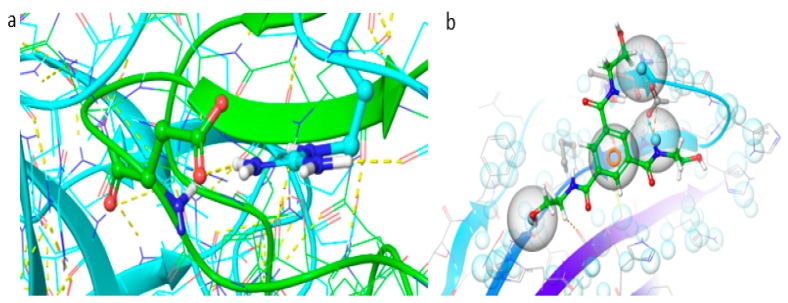
(**a**) Representation of main interactions between Asp298 (tyrosine kinase receptor B: TrkB) —green and Arg11 (neurotrophin (NT)-4/5)—blue; (**b**) representation of the developed pharmacophore hypothesis with its features.

After conducting in silico studies on the crystal structure of the TrkB receptor in order to determine the most important interactions between TrkB and its natural ligand—neurotrophin-4/5 (NT-4/5) (Figure 20a)—we performed in silico screening of the unique CERMN (Centre d’Etudes et de Recherche sur le Médicament de Normandie) library of compounds. The screening was done on the basis of the pharmacophore hypothesis (Figure 20b) developed from interactions of TrkB and its synthetic ligand—LM22A-4 (Massa, S.M., et al. *J. Clin. Invest.* **2010**, *120*, 1774–1785). On the basis of the main structural features of the LM22A-4, we designed a small dataset of the new potential TrkB agonists and conducted the synthesis of the compounds with best in silico results.

This project received funding from the European Union′s Horizon 2020 framework programme for research and innovation under grant agreement no. 765704.

### 7.27. Isolation and Structure Elucidation of Aspergillus Metabolites Induced with Epigenetic Modulators (P27)

AldholmiMohammed^1^,^2^GanesanA.^1^1School of Pharmacy, University of East Anglia, Norwich Research Park, Norwich NR4 7TJ, UK2Natural Products and Alternative Medicine, College of Clinical Pharmacy, Imam Abdulrahman Bin Faisal University, Dammam 31441, Saudi Arabia; m.aldholmi@uea.ac.uk

*Aspergillus* species are rich source of bioactive metabolites. However, most natural product biosynthetic pathways are silent under laboratory conditions. The epigenetic processes, including the covalent modifications of DNA and amino acids on histones, have been revealed to be extensively used by organisms to regulate the expression of genes involved in natural product biosynthesis (Felsenfeld, G., et al. *Nature* **2003**, *421(6921)*, 448–453; Cichewicz, R. H. et al. *Nat. Prod. Rep.* **2010**, *27(1)*, 11–22). Therefore, it is possible to influence these processes during microbial fermentation by the addition of chemical epigenetic modulators (Cichewicz, R. H, et al. *Nat. Prod. Rep*. **2010**, *27(1)*, 11–22; Williams, R.B., et al. *Org. Biomol. Chem*. **2008**, *6(11)*, 1895–1897).

Five *Aspergillus* strains were cultivated in the presence of Histone Deacetylase Activity (HDAC) inhibitors (e.g., vorinostat) or DNA methyltransferase (DNMT) inhibitors (e.g., 5-azacytidine). Morphological changes were monitored for 7 days before extraction with ethyl acetate or methanol. The extracts were analysed by HPLC and LC-MS, and the metabolic profiles were compared using chromatogram overlay and volcano plots.

The overlayered chromatograms and volcano plots presented significant differences between the cultures treated with epigenetic modifiers and control cultures. Two induced bioactive metabolites were isolated from *Aspergillus westerdijkiae* and *Aspergillus calidoustus*, and their structures were elucidated using mass spectrometry and NMR.

I would like to thank Imam Abdulrahman Bin Faisal University for their financial support.

### 7.28. Pharmacomodulation of Antiplasmodial Trichloromethylated-Heterocycles Using Ring Variation Strategy (P28)

AmraneDyhia^1^PrimasNicolas^1^HutterSébastien^2^AzasNadine^2^VerhaeghePierre^3^VanellePatrice^1^1Aix-Marseille Univ, CNRS, ICR UMR 7273, PCR, Faculté de Pharmacie, 13385 Marseille2Aix-Marseille Univ, IHU Méditerranée Infection, UMR VITROME, 13005 Marseille3Université Paul Sabatier, CNRS UPR 8241, LCC, 31077 Toulouse; nicolas.primas@univ-amu.fr

**Figure 21:**
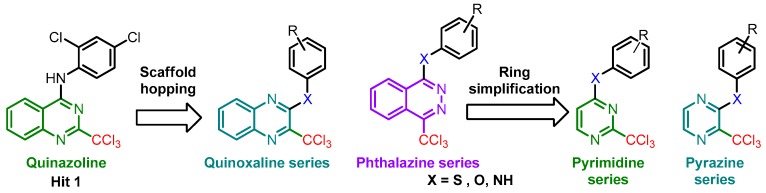
Ring variation strategy adopted in order to explore SARs.

Malaria is still the leading cause of death among parasitic infections worldwide (WHO, World Malaria report, **2018**, http://www.who.int/malaria/publications/world-malaria-report-2018/report/en/). The emergence and the expansion of *Plasmodium* strains resistant to the artemisinin-based combination therapies (ACTs) are now threatening the efficacy of malaria treatment, notably in the Greater Mekong sub-region. Therefore, new molecules displaying the original mode of action are urgently required. Aiming at developing new antiplasmodials, our laboratory previously described the synthesis and the biological activities of a library of 2-trichloromethylquinazoline derivatives, which highlighted a hit molecule 1 (IC_50_ = 0.4 µM, CC_50_ = 16 µM) (Cominetti, M. D. D., et al. *Org. Biomol. Chem.* **2016**, *14*, 10161–10164; Verhaeghe, P., et al. *Bioorg. Med. Chem.* **2009**, *17*, 4313–4322) (Figure 21). Moreover, a scaffold-hopping strategy showed that the replacement of the quinazoline moiety by a quinoxaline one improved the cytotoxicity profile (Desroches. J., et al. *Eur J. Med. Chem.* **2017**, 125, 68–86). Thus, we synthetized a new series of 2-trichloromethylquinoxaline analogues. The in vitro biological evaluations against the multi-resistant K1 *P. falciparum* strain highlighted two new hit molecules substituted by an electron-withdrawing group in *para*-position. Accordingly with these results, we prepared new 2-trichloromethylquinoxaline analogues bearing other electron-withdrawing groups such as -CF_3_, -SF_5_ or -OCF_3_.

In parallel, to complete the global SAR study using the scaffold hopping approach, we replaced the quinoxaline ring with a phthalazine one. The importance for the antiplasmodial activity of the phenyl ring contained in the bicyclic quinazoline and quinoxaline scaffolds was also studied by structural simplification, leading to the related pyrimidine and pyrazine analogues. The synthesis details of the new analogues of hit 1 in various series and the biological results will be described in the poster.

This work was supported by the ANR NINTARMAL project, grant ANR-17-CE11–0017.

### 7.29. Optimisation of Peptide Linker-Based Fluorescent Ligands for Histamine H1 Receptor (P29)

KokZhi Yuan^1^MistryShailesh N.^1^HillStephen J.^2^KellamBarrie^1^1School of Pharmacy, University of Nottingham, NG7 2RD, UK2School of Life Sciences, University of Nottingham, Nottingham NG7 2UH, UK; paxzyk@nottingham.ac.uk

The histamine H_1_ receptor (H_1_R) is a class A GPCR and is widely expressed throughout the human body. H_1_R-dependent signalling is responsible for allergic reactions, plays an important role in immunomodulation and, as recent studies have suggested, is involved in mediating cell proliferation and thus cancer progression (Panula, P., et al. *Pharmacol Rev.* **2015**, *67* (3), 601–655). High affinity H_1_R fluorescent ligands with optimum physicochemical properties would serve as a valuable tool to probe in further detail H_1_R cancer pharmacology and drug discovery (McGrath, J. C., et al. *The Adrenergic Receptors*, Springer: **2006**; pp 151–172; Vernall, A. J., et al. *British J. Pharmacol.* **2014**, *171* (5), 1073–1084). Fluorescent ligands comprise a receptor binding element (either an orthostere or allostere) linker connect to an appropriate fluorophore. Whilst modulating the overall physicochemical properties of the fluorescent ligand, a peptide-based linker could engage in receptor binding through its side chain functional groups, potentially improving binding affinity of the overall conjugate and providing information on SAR along the receptor exit tunnel. The crystal structure of H_1_R in complex with the antagonist doxepin was reported in 2011, providing detailed information of the orthosteric binding site and revealing an additional binding site lined by the basic amino acids of Lys179, Lys191 and His450, which could be targeted to improve ligand affinity (Shimamura, T., et al. *Nature* **2011**, *475* (7354), 65–70).

We envisaged optimising the peptide linker for a previously reported H_1_R fluorescent ligand (Stoddart, L. A., et al. *Scientific Reports* **2018**, *8 (1)*, 1572) with pK_D_ of 8.1 by sequential screening for amino acids along the peptide chain in order to identify those that could provide the greatest improvement in binding affinity. By incorporating these amino acids into the linker, we synthesised H_1_R fluorescent ligands with pK_D_′s ranging from 7.8 to 8.4. Molecular docking of these ligands into an H_1_R crystal structure suggested the existence of small binding pockets along the receptor exit tunnel that were exploited by the functionalised peptide linkers. Further experimentation with fluorescent ligands of varying fluorophores suggested that the attachment of the boron-dipyrromethene BODIPY630/650 fluorophore was the main driving force in binding affinity in H_1_R fluorescent ligands. Despite not being able to significantly improve binding affinities of H_1_R fluorescent ligands, optimisation of the peptide linker provided information on SAR along the receptor exit tunnel.

### 7.30. First Design of MT5-MMP Inhibitors, a Challenging New Target for Alzheimer′s Disease (P30)

ZipfelPauline^1^LalutJulien^1^DenisCamille^1^BureauRonan^1^SuzannePeggy^1^DavisAudrey^1^Malzert-FréonAurélie^1^BarangerKevin^2^KhrestchatiskyMichel^2^RiveraSantiago^2^RochaisChristophe^1^DallemagnePatrick^1^1Centre d′Etudes et de Recherche sur le Médicament de Normandie (CERMN), Normandie Univ, UNICAEN, 14000 Caen, France2Institute of Neuropathophysiology (INP), UMR7051, CNRS, Aix Marseille Université, France; pauline.zipfel@unicaen.fr

In 2018, the number of people living with dementia in the world was estimated at 50 million, and Alzheimer′s disease (AD) is the most common form of dementia. AD is a neurodegenerative and incurable brain disorder; only treatments for symptoms are available at this time. Because of the heavy economic and societal impacts, there is an urgent need to find new treatments that target the molecular causes of neuronal cell death. Two abnormal structures in the brain called β-amyloid (Aβ)-containing plaques and neurofibrillary tangles are considered as two of the main features of AD. In this context, several studies support the hypothesis that alterations in the processing of amyloid precursor protein (APP) contributes to AD pathogenesis, resulting in the accumulation of β-amyloid peptides (Aβ) and other proteolytic products. Thus, current research focuses on the enzymes involved in APP cleavage such as α-, β- and γ-secretases. However, recent studies have revealed the existence of another physiological APP processing pathway, mediated by a novel AD-related enzyme, membrane-type 5-matrix metalloproteinase (MT5-MMP), which can process APP and promote Aβ accumulation, as well as the inflammatory process in AD transgenic mice (Baranger, K., et al. *Cell. Mol. Life Sci.* **2016**, *73*(1), 217–236); Baranger, K. et al. *J. Neuroinflammation* **2016**, *13*(1), 167–170); Baranger, K., et al., *Front. Mol. Neurosci.* **2017**, *9*, 1–17). Moreover, MT5-MMP can cleave APP upstream from the β-secretase cleavage site (the so called η-cleavage site) (Ahmad, M. *et al., J. Biochem.* **2006**, *139*(3), 517–526) and release an N-terminally elongated Aβ fragment (Aη-α), which appears to be synaptotoxic (Willem, M., et al. *Nature* **2015**, *526(7573)*, 443–447). We aim to design and synthesize first selective MT5-MMP inhibitors through an interdisciplinary approach including molecular modelling, medicinal chemistry and biology. Starting from a cyclopentathiophene derivative identified by in silico screening, we are currently investigating the pharmacomodulations on this hit compound to gain in affinity and selectivity for MT5-MMP.


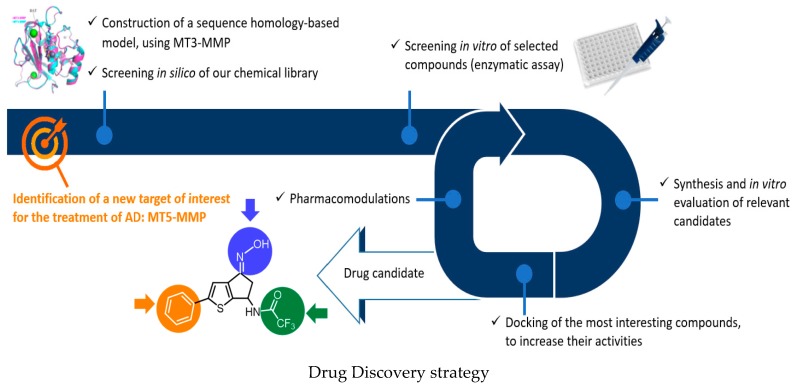


### 7.31. Design, Synthesis and Evaluation of Quinolines for Drug-Resistant Malaria (P31)

MagwazaRachael^1^HusseinButhaina^1^AbubakerMuna^2^FreemanSally^1^NirmalanNiroshini^2^1Department of Pharmacy and Optometry, University of Manchester, Manchester M13 9PL, UK2Department of life sciences, University of Salford, Salford M5 4WT, UK; rachael.magwaza@manchester.ac.uk

Malaria is an infectious disease caused by the parasite of the genus *Plasmodium* with human infection caused by species *falciparum*, *malaria*, *knowlesi*, *vivax* and *ovale* (De Koning-ward, T. F., et al. *Nat. Publ. Gr*. **2016**, 14, 494–507). *P. falciparum* is associated with the most severe form of malaria, responsible for ≈400,000 deaths/year, especially in Africa. A number of antimalarial drugs are currently used to treat malaria. However, *P. falciparum* has developed resistance to all currently used medicines, hence, there is a great need for the development of new drugs. Here, we report novel quinolines as antimalarial leads. The quinolines were originally designed to be inhibitors of NRH—quinone oxidoreductase 2 (NQO2), a potential therapeutic target in cancer chemotherapy (Alnabulsi, S., et al. *Bioorg. Med. Chem. Lett*. **2018**; 9, 1–6). *P. falciparum* contains a functionally similar type II NADH/dehydrogenase known as *Pf*NDH2; therefore, these compounds were tested against *Plasmodium falciparum* as antimalarial leads. The quinolines were identified as a potent in vitro inhibitor of the multi-drug-resistant strain K1 of *P. falciparum* (with EC_50_ ranging from 0.5–20 µM). The EC_50_ speed assays carried out to verify onset of antimalarial activity showed that the compounds were fast-acting, as the EC_50_ values were achieved at 24 h. Cell cytotoxicity assays showed that the compounds were very toxic towards HepG2 hepatic cancer cell lines seeded at 4000 cells/well, with low selectivity indices (SI 0.6–2.7). The compounds were screened further against MDBK bovine kidney cells, which showed improved selectivity indices (SI 1.6 > 10). To achieve further dose reduction and to minimize toxicity, Calcusyn-based drug interactivity assays are ongoing. Further studies are in progress to characterize stage specificity, mechanism of action, cytotoxicity profiles and drug interaction with existing antimalarial drugs.

### 7.32. Design, Synthesis and Biological Evaluation of New Fluorinated Indazole Derivates as Potential New 5-HT_4_R PET Radiotracers (P32)

MangeantReynald^1^StiebingSilvia^1^CaillyThomas^1^,^2^DavisAudrey^1^FabisFrederic^1^CollotValerie^1^1Normandie univ, UNICAEN, CERMN, 14000 Caen, France2Department of Nuclear Medecine, CHU Côte de Nacre, 14000 Caen, France; reynald.mangeant@unicaen.fr

**Figure 22:**
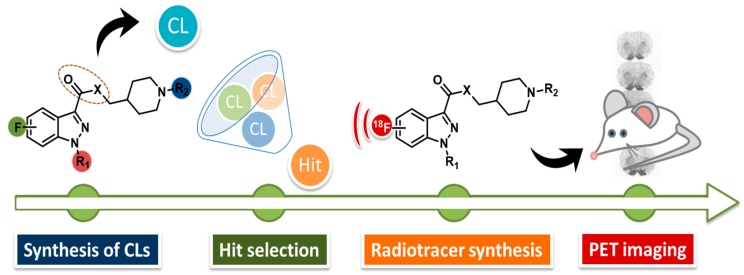
Essential steps to obtain radiotracers. CL: cold ligands, PET: positron emission tomography.

Since its discovery in 1988, the serotonin 4 receptor subtype (5-HT_4_R) has emerged as a promising target for drug discovery and development, resulting from their implications in learning, cognition, memory processes and many neuropsychiatric disorders such as Alzheimer′s disease, anxiety, depression or anorexia nervosa (Bockaert, J., et al. *Neuropharmacology* **2008**, *55 (6)*, 922–931). Thus, discovery of active 5-HT_4_R agonists and antagonists remains a continuing interest in clinical research. To this end, positron emission tomography (PET) (Marner, L., et al. *Neuroimage* **2010**, *50* (3), 855–861; Caillé, F., et al. *Bioorg. Med. Chem. Lett.* **2013**, *23* *(23)*, 6243–6247) coupled with effective radioligands constitutes a valuable tool, both in clinical studies and drug discovery programmes. On the basis of previous works in CERMN (Lam, B. V., et al. *Chem. Eur. J.* **2016**, *22* (13), 4440–4446), we aimed to develop new fluorinated indazole derivates as potential brain 5-HT_4_R PET tracers (Figure 22).

This synthesis was carried out in three essential steps. A convergent synthesis pathway to obtain cold ligands (CL) was established. A methodology allowing selective functionalization at position 3 leading to polyfunctional indazoles in a few steps was developed. New pharmacomodulations studies were realized in order to increase receptor affinity, decrease lipophilicity and increase metabolic resistance.

More than 20 compounds with nanomolar affinity were synthesized. One of them, MR35806, is currently radiolabeled with fluorine 18 to obtain our first brain 5-HT_4_R PET tracer. This compound will be tested in vivo on mouse brain.

The authors are grateful for the financial support by regional council of Normandy, FEDER and Crunch Network.

### 7.33. Acetoxystachybotrydial Acetate, a Natural Compound Isolated from Stachybotrys Chartarum is a Potent Inhibitor of Human Protein Casein Kinase 2 (CK2) *(P33)*

HaidarSamer^1^JürgensFranziska M^2^AicheleDagmar^1^JagelsAnnika^2^HumpfHans-Ulrich^2^JoseJoachim^1^1Institut für Pharmazeutische und Medizinische Chemie, PharmaCampus, und 2Institut für Lebensmittelchemie, Westfälische Wilhelms-Universität Münster, 48149 Münster, Germany2Institute of Food Chemistry, Westfälische Wilhelms-Universität Münster, 48149 Münster, Germany; shaid_01@uni-muenster.de

**Figure 23:**
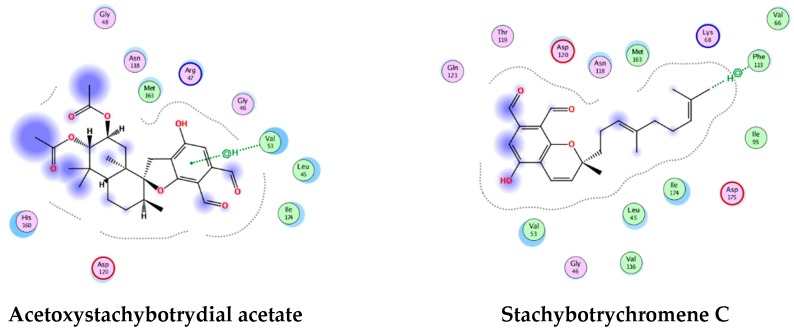
2D representation of the interaction between the ATP binding site of CK2 with acetoxystachybotrydial acetate and stachybotrychromene C.

Human protein kinase CK2 is an emerging target for drug design. Overexpression of CK2 is closely related to many types of human cancer. It has been shown that elevated levels of CK2 protect tumour cells from apoptosis (Faust M, Montenarh M. *Cell Tissue Res* **2000**, *301*, 329–340). Inhibition of CK2 activity can lead tumour cells into apoptosis, whereas viability of healthy cells is not affected (Ruzzene, M., et al. *Biochim. Biophys. Acta* **2010**, *1804*, 499–504). Up until now, one ATP competitive inhibitor of CK2, silmitasertib, is in phase-II clinical trials (Gowda, C., et al. *Curr. Pharm. Des.* **2017**, *23*, 95–107). Here, we report on the screening of natural compounds isolated from *Stachybotryschartarum* (Gratz, A., et al. *Electrophoresis* **2010**, *31*, 634–640). Twelve phenylspirodrimanes and three triphenylphenoles were investigated on inhibitory activity towards CK2 using a capillary electrophoresis-based assay (Jagels, A., et al. *Mycotoxin Res.* **2018**, 34, 179–185). Triphenyl phenolestachybotrychromene C, phenylspirodrimanes stachybotrydial acetate and acetoxystachybotrydial acetate with IC_50_ values of 0.3 µM, 0.7 µM and 1.9 µM, respectively, were identified as potent CK2 inhibitors (Figure 23). The effect of these compounds on the proliferation of breast cancer cells MCF-7 was determined using an EdU (5-ethynyl-2’-deoxyuridine) assay. For comparison, viability of breast cancer cells is shown below.

Cancer cells MCF-7 as well as lung cancer cells A427 and epidermal cancer cells A 431 were tested using MTT assay. In particular, acetoxystachybotrydial acetate turned out to be the most active compound. After treatment of MCF-7 cells with 1 µM for 24 h, cell proliferation was blocked almost completely (99%), whereas cell viability was decreased only by 63%. Here, we describe phenlyspirodrimanes as a new class of CK2 inhibitors, among them acetoxystachybotrydial acetate which has been proven to be the most potent representative of this series.

### 7.34. Synthesis and Biological Evaluation of New Indolo [2, 3-b] Quinoline Derivatives as Potential Antimalarial Candidates (P34)

DoudetLudovic^1^Allart-SimonIngrid^1^MaciukAlexandre^2^SapiJanos^1^GérardStéphane^1^1Université de Reims Champagne-Ardenne, Institut de Chimie Moléculaire de Reims (ICMR), UMR CNRS 7312, UFR Pharmacie, 51 rue Cognacq-jay, 51096 Reims, France2Université de Paris Sud, BioCIS UMR CNRS 8076, UFR Pharmacie, 92296 Châtenay-Malabry, France; stephane.gerard@univ-reims.fr

**Figure 24:**
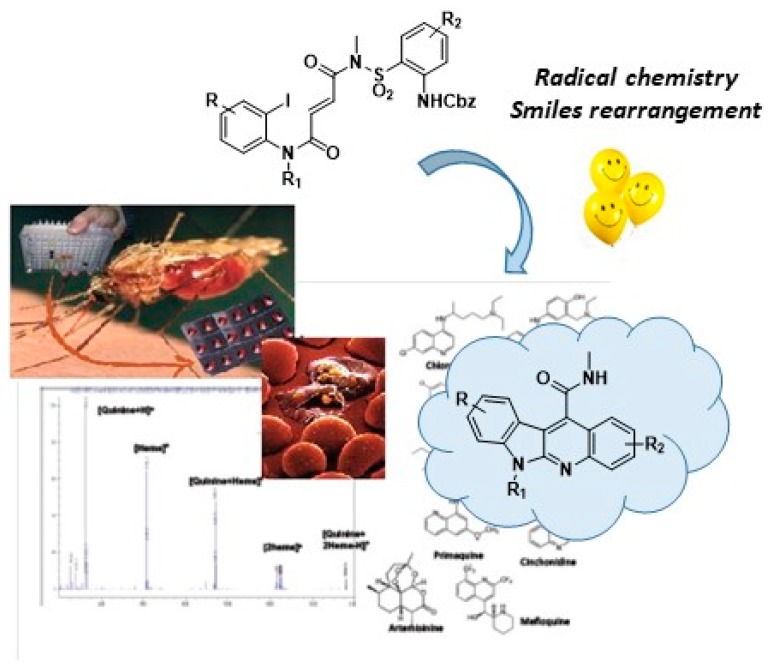
Strategy for the synthesis of a variety of heterocyclic compounds.

The indolo [2, 3-*b*] quinoline system is present in many marine alkaloids that have demonstrated interesting biological activity (for a review on biological activities, see: Lavrado, J., et al. *Curr. Med. Chem.* **2010**, *17*, 2348–2370). Smiles rearrangement, an organic intramolecular nucleophilic aromatic substitution reaction, provides a powerful access to the synthesis of a variety of heterocyclic compounds (Figure 24). Taking advantage of the combination of Smiles rearrangement and radical chemistry, preparation of new indolo [2, 3-*b*] quinoline derivatives and their biological evaluation will be presented (Allart-Simon, I., et al. *Molecules* **2016**, *21*, 878–889; Pudlo, M., et al. *Chem. Comm.* **2012**, *48*, 2442–2444).

### 7.35. Isobile Acids, Their Toxicity and Effect on Activation of farnesoid X receptor (FXR) (P35)

LuYin^1^WielSandra Van de^2^GilmerJohn^1^1School of Pharmacy and Pharmaceutical Sciences, Trinity College Dublin, D02 PN40 Dublin 2, Ireland2Tytgat Institute for Liver and Intestinal Research, Department of Gastroenterology and Hepatology, Amsterdam Gastroenterology and Metabolism, Academic Medical Center, Amsterdam, The Netherlands; yilu@tcd.ie

**Figure 25:**
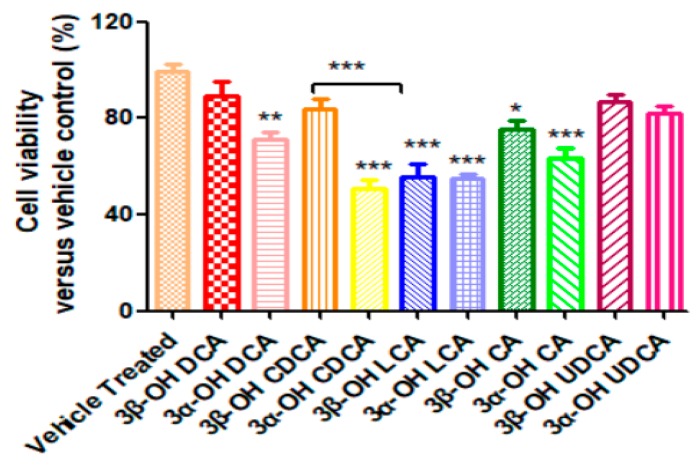
A measurement of toxicity of different bile acids. Caco-2 cells were treated with 100 μM bile acid solution for 24 h, and cells were then treated with 10 μl of 2.5 mg/ml MTT reagent and incubated for 2 h.

**Figure 26:**
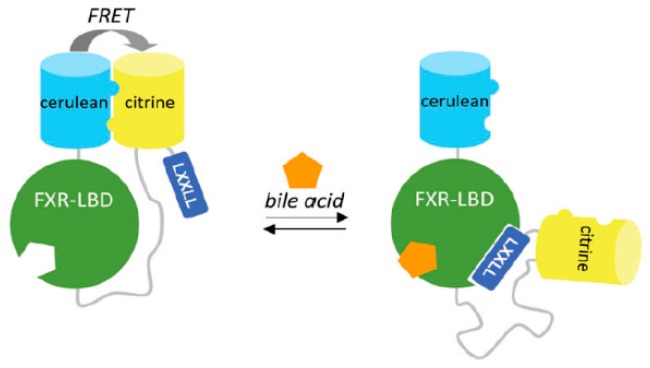
Schematic representation the mechanism of BAS upon FXR activation.

Bile acids have conventionally been thought of as mere digestive surfactants that assist in the absorption of fats and fat soluble vitamins. In the past two decades, it has emerged that bile acids play important functions as signalling molecules in mammalian physiology and that, for example, they can regulate bile acid metabolism. FXR, a member of nuclear receptor superfamily, plays a crucial role in regulating bile acid homeostasis (Kullak-Ublick, G.A., et al. *Physiology*. **2008**, *23(5)*, 286–295) and an abundance of evidence suggests that FXR may be a useful target in the prevention of colon carcinogenesis (Modica, S., et al. *Cancer Res*. **2008**, *23*, 9589–9595). It can be hypothesised that isobile acids (the 3β-OH isomers of the normally occurring bile acids) can activate the FXR and regulate FXR function through their presence in the bowel and isobile acids can modify the toxicity of the bile acids (Figure 25 and Figure 26).

We explored new synthetic approaches for producing isobile acids from natural bile acids, and a panel of isobile acids and normal bile acids were prepared in order to help in the characterization of their effect on cellular toxicity and on FXR activation.

We reached the conclusion that among the natural bile acids, CDCA was found to be the most toxic, followed by LCA, CA and DCA. UDCA was least toxic, and this was as expected from many studies on the relative toxicity of the bile acids, corresponding to the order of hydrophobicity of the bile acids. In general, the beta compounds (isobile acids) were less toxic than the alpha compounds. Only in the case of LCA was there no difference in the toxicity of the epimeric pairs. Only isoCDCA was found as a true FXR agonist on the basis of FXR FRET assay, whereas CDCA showed no significant effect in FRET, which was surprising.

We are most grateful to Dr. John O′Brien for NMR spectroscopy, Gary Hessman and Brian Talbot for MS spectroscopy and Tarek Moustafa for his collaboration in the FXR FRET assay.

### 7.36. Synthesis and Biological Activities of New 2-Mercapto-(2-Oxoindolin-3-ylidene)acetonitrile Derivatives (P36)

LetribotBorisDelatoucheRégisChérouvrierJean-RenéDomonLisianneThiéryValérieLa Rochelle UniversityLIENSs UMR 7266, 17000-F La Rochelle, France; valerie.thiery@univ-lr.fr

**Figure 27:**
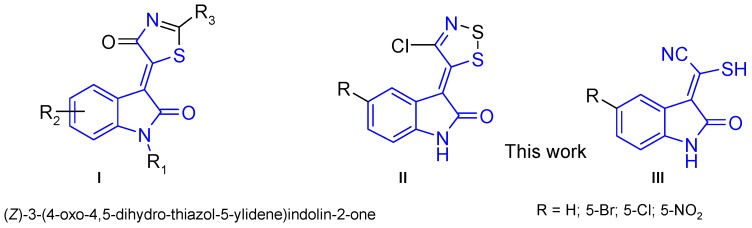
The (*Z*)-3-(4-oxo-4,5-dihydro-thiazol-5-ylidene)indolin-2- one pattern **I** and targets synthesis 3-(4-chloro-5*H*-1,2,3-dithiazol-5-yliden)indolin-2-one **II** and (2-oxoindolin-3-ylidene)-2-mercaptoacetonitrile **III**.

The occurrence of the 3-alkenyl-oxindole skeleton in various natural and synthetic products has generated interest of many groups on account of its useful biological properties and its versatility as a useful intermediate in the synthesis of many compounds (Bort, A., et al. *Sci. Rep.* **2018**, *8*, 4370). As part of our ongoing research on oxindole derivatives, we previously designed, synthesized and evaluated new series of bis-oxindoles as potent kinase inhibitors (Beauchard, A., et al. *Bioorg. Med. Chem.* **2006**, *1*, 6434–6443). In an effort to identify new pharmacological inhibitors of disease-relevant protein kinases with increased potency and selectivity, we launched a research program dealing with the synthesis of rare substituted 2-(2-oxoindolin-3-ylidene)acetonitrile derivatives bearing a sulphur atom directly attached on the external double bond. Few 3-(thiazol-5-ylidene)indoline-2-one derivatives **I** have been reported in the literature (Havrylyuk, D., et al. *J. Med. Chem*. **2012**, 55, 8630–8641; Erben, F., et al. *RSC Adv*. **2014**, *4 (21)*, 10879–10893). To access the required 2-oxoindolylacetonitrile derivatives **III** (Figure 27), we decided to explore a new route based on the application in synthesis of the reagent 4,5-dichloro-1,2,3-dithiazolium chloride (Appel salt) **1**. The work described in this poster represents further application of Appel salt in the conception of novel heterocyclic rings to access to original bioactive compounds.

Thanks to the “Ligue Nationale Contre le Cancer, comité 17” for financial support and to the “Conseil Départemental de Charente-Maritime” for PhD grant (BL).

### 7.37. Building a Diverse and Experimentally-Curated Fragment Library (P39)

BroughSteve^1^LowersonAndrew^1^LaPlanteSteven^2^McCarrenPatrick^3^Serrano-WuMichael^3^1Key Organics Limited, Cornwall, UK2NMX Research and Solutions, Montreal, CA3Broad Institute, Cambridge, MA, USA; steveb@keyorganics.net

Fragment libraries are commonly assembled by rule of three filtering followed by manual curation. However, the robust experimental data that ensures the proper physicochemical attributes needed for high-concentration screening is often lacking and replaced instead by in silico calculations of uncertain predictive value. A fragment collection with experimentally-determined aqueous solubility will address a major source of false positives and attrition in fragment screening libraries: aggregation, stability and solubility. ^1^H NMR spectral data in aqueous buffer will further enable practitioners to rapidly build fragment pools and initiate screening.

Diversity selection methods in shape, scaffold, fingerprint and predicted property space combined with industry-standard substructure filtering were used to select over 2500 Key Organics compounds for experimental profiling. NMR and LC–MS analysis allowed the careful selection of highly-soluble fragments with desirable physicochemical and stability characteristics. Importantly, the curated molecules were enriched in cyclic scaffolds commonly found in drug candidates and spanned chemical space that minimally overlapped with existing commercial collections. This poster summarizes the experimental and cheminformatic features of this next generation Key Organics ‘BIONET Premium Fragment Library′. (Bradley, C., et al. *J. Chem. Inf.Model.* **2006**, *46*, 1060–1068; Baell, J. B., et al. *J. Med. Chem.* **2010**, *53*, 2719–2740; Lagorce, D., et al. *BMC Bioinformatics* **2008**, *9*, 396; Kazius, J., et al. *J. Med. Chem.* **2005**, *48*, 312–320; Bruns, R. F., et al. *J. Med. Chem.* **2012**, *55*, 9763–9772; Lisurek, M, et al. *Mol. Divers.* **2010**, *14(2)*, 401–408; Taylor, R. D., et al. *J. Med. Chem.* **2014**, *57 (14)*, 5845–5859).


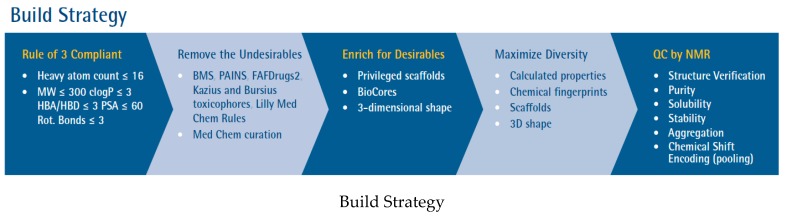


### 7.38. Overcoming tumor necrosis factor (TNF)-Related Apoptosis Inducing Ligand (TRAIL) Resistance in Cancer Stem Cells: Computer-Aided Discovery of c-FLIP Inhibitors

LeeKok Yung^1^BrancaleAndrea^1^ClarksonRichard^2^WestwellAndrew^1^1School of Pharmacy and Pharmaceutical Sciences, Cardiff University, Cardiff CF10 3NB, UK2European Cancer Stem Cell Research Institute, Cardiff University, Cardiff CF24 4HQ, UK; leek10@cardiff.ac.uk

**Figure 28:**
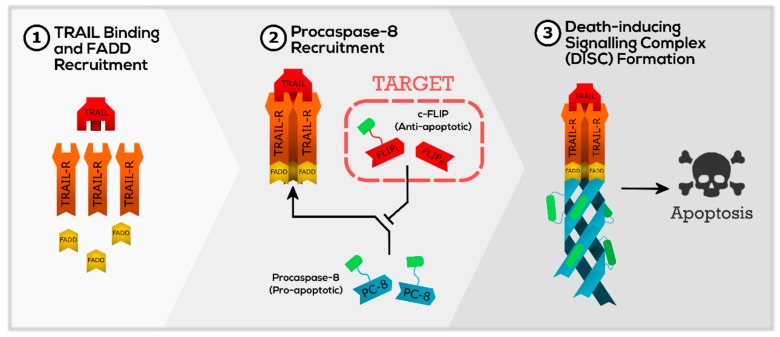
The role of c-FLIP in the extrinsic apoptotic pathway.

The ability of cancer stem cells to self-renew indefinitely and differentiate into multiple tumour cell types has made their elimination critical to completely eradicate tumours (Battle, E.; Clevers, H. *Nat Med*. **2017**, *23* (10), 1124–1134). TRAIL (TNF-related apoptosis inducing ligand) is a death ligand that selectively induces apoptosis in cancer cells, but clinical trials utilising recombinant TRAIL or TRAIL agonists have eventually failed due to the development of TRAIL resistance post-treatment (de Miguel, D., et al. *Cell Death Differ*. **2016**, *23 (5)*, 733–747; Herbst, R. S.; et al. *J Clin Oncol*. **2010**, *28 (17)*, 2839–46)(Figure 28). c-FLIP (cellular FLICE-like inhibitory protein) inhibits TRAIL-mediated apoptosis and its overexpression has since been identified as one of the mechanisms of TRAIL resistance present in cancer stem cells (French, R.; et al. *Mol Cancer*. **2015**, *14*, 209). As a strategy to overcome TRAIL resistance, this project aims to develop new small molecule inhibitors of c-FLIP that re-sensitises cancer stem cells to TRAIL-mediated apoptosis.

Twenty different compounds were identified through in silico screening as potential lead compounds targeting DEAD-box protein 1 (DED1) of c-FLIP. HeLa cells were treated with these compounds in combination with TRAIL to test for their ability to sensitise HeLa cells to TRAIL-mediated apoptosis. Caspase-mediated apoptosis was confirmed through rescue with a pan-caspase inhibitor. Three of twenty compounds in combination with TRAIL successfully induced apoptosis in HeLa cells rescuable by caspase inhibitor. Further work is in progress to confirm c-FLIP inhibitor activity of these compounds through co-immunoprecipitation while analogues of the best performing lead compound are synthesised.

### 7.39. University of Nottingham Managed Chemical Compound Collection (MCCC) (P41)

HashmiLubnaFischerPeterStocksMichaelCentre for Biomolecular Sciences, University of Nottingham, Nottingham, Nottinghamshire NG7 2RD, UK; michael.stocks@nottingham.ac.uk

*An Affordable and Automated Compound Management Facility*

• Compound library for high and medium throughput screening;
• >85K Structurally diverse drug-like molecules;
○ Molecules obey Lipinski “rule of 5”;
○ No reactive functional groups.

*Purity Analysis and Structural Confirmation*

- Samples stored at optimum condition as 10mM DMSO stock solutions;
  - Dedicated LC–MS system to ensure purity of compounds.

*Medicinal and Computation Chemistry Support*

- Collaborative projects welcome.

*For further information please contact:*

Dr Michael Stocks; Head of Division of Medicinal Chemistry and Structural Biology; michael.stocks@nottingham.ac.uk

## 8. Conclusions

The meeting attracted 114 delegates with successful growth of both the network membership and the geographical range of attendee home countries, including France, Germany, Russia, Spain, Portugal, Ireland, Italy and the United Kingdom. The programme comprised a discipline-leading line-up of presenters from both academic and industrial sectors (including talks from 2 plenaries, 12 keynotes and 6 young researchers, and 41 posters), spanning a broad range of topics related to medicinal chemistry. For the first time, the inclusion of a pre-conference workshop expanded attendee knowledge and skills in the area of quantitative pharmacology. In addition, attendees of the conference dinner enjoyed some words from Emeritus Prof. Malcolm Stevens FRS (UoN), who appropriately wore a bowtie dyed with the original mauveine made by William Henry Perkin.

A number of prizes were awarded, with the best poster prize (sponsored by the Royal Society of Chemistry) going to Mirjana Antonijevic (UNICAEN, France), the best young researcher oral presentation to Scott Grossman (School of Pharmacy, University of Nottingham) and the MDPI *Pharmaceuticals* travel award to Jorge Grilo (University of Lisbon).

The 28th Annual GP2A Medicinal Chemistry Conference will take place between 26 and 28 August 2020 at the University of La Rochelle, France.

The GP2A committee and the local organizing committee thank the Division of Biomolecular Science and Medicinal Chemistry, School of Pharmacy (UoN), and all the sponsors and exhibitors for supporting this annual event.

## Figures and Tables

**Table 1:** General structure of the compounds.

Compound	R_1_	R_2_	R_3_
Phloretin (Pt)	OH	OH	H
Phlorizin	OH	OGlu	H
3-Hydroxyphloretin (HPt)	OH	OH	OH
Trilobatin	OGlu	OH	H
Sieboldin	OGlu	OH	OH

